# Identification of a novel monocyclic carotenoid and prediction of its biosynthetic genes in *Algoriphagus* sp. oki45

**DOI:** 10.1007/s00253-023-12995-2

**Published:** 2024-01-11

**Authors:** Naoki Takatani, Takashi Maoka, Tomoo Sawabe, Fumiaki Beppu, Masashi Hosokawa

**Affiliations:** 1 Faculty of Fisheries Sciences, Hokkaido University, 3-1-1 Minato, Hakodate, Hokkaido 041-8611 Japan; 2 Research Institute for Production Development, 15 Shimogamo-Morimoto-Cho, Sakyo-Ku, Kyoto, 606-0805 Japan

**Keywords:** *Algoriphagus*, Monocyclic carotenoids, Flexixanthin, 2-Hydroxyflexixanthin, Carotenoid biosynthetic genes, 2,2ʹ-β-Hydroxylase

## Abstract

**Abstract:**

Bacteria belonging to the genus *Algoriphagus* have been isolated from various sources, such as Antarctic sea ice, seawater, and sediment, and some strains are known to produce orange to red pigments. However, the pigment composition and biosynthetic genes have not been fully elucidated. A new red-pigmented *Algoriphagus* sp. strain, oki45, was isolated from the surface of seaweed collected from Senaga-Jima Island, Okinawa, Japan. Genome comparison revealed oki45’s average nucleotide identity of less than 95% to its closely related species, *Algoriphagus confluentis* NBRC 111222^ T^ and *Algoriphagus taiwanensis* JCM 19755^ T^. Comprehensive chemical analyses of oki45’s pigments, including ^1^H and ^13^C nuclear magnetic resonance and circular dichroism spectroscopy, revealed that the pigments were mixtures of monocyclic carotenoids, (3*S*)-flexixanthin ((3*S*)-3,1′-dihydroxy-3′,4′-didehydro-1′,2′-dihydro-β,ψ-caroten-4-one) and (2*R*,3*S*)-2-hydroxyflexixanthin ((2*R*,3*S*)-2,3,1′-trihydroxy-3′,4′-didehydro-1′,2′-dihydro-β,ψ-caroten-4-one); in particular, the latter compound was new and not previously reported. Both monocyclic carotenoids were also found in *A. confluentis* NBRC 111222^ T^ and *A. taiwanensis* JCM 19755^ T^. Further genome comparisons of carotenoid biosynthetic genes revealed the presence of eight genes (*crtE*, *crtB*, *crtI*, *cruF*, *crtD*, *crtYcd*, *crtW*, and *crtZ*) for flexixanthin biosynthesis. In addition, a *crtG* homolog gene encoding 2,2ʹ-β-hydroxylase was found in the genome of the strains oki45, *A. confluentis* NBRC 111222^ T^, and *A. taiwanensis* JCM 19755^ T^, suggesting that the gene is involved in 2-hydroxyflexixanthin synthesis via 2-hydroxylation of flexixanthin. These findings expand our knowledge of monocyclic carotenoid biosynthesis in *Algoriphagus* bacteria.

**Key points:**

*• Algoriphagus sp. strain oki45 was isolated from seaweed collected in Okinawa, Japan.*

*• A novel monocyclic carotenoid 2-hydroxyflexixanthin was identified from strain oki45.*

*• Nine genes for 2-hydroxyflexixanthin biosynthesis were found in strain oki45 genome.*

**Graphical Abstract:**

**Figure:**
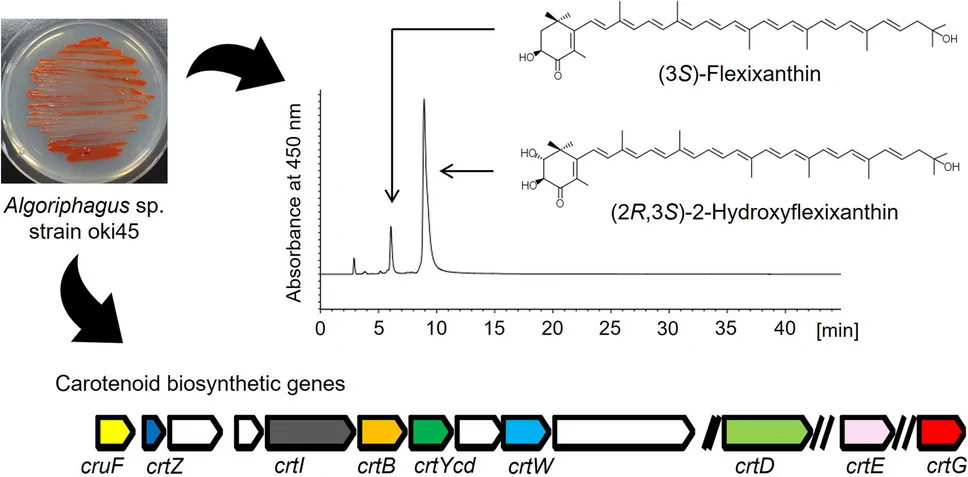

**Supplementary Information:**

The online version contains supplementary material available at 10.1007/s00253-023-12995-2.

## Introduction

Carotenoids are red-to-yellow lipophilic pigments biosynthesized by plants and microorganisms, and > 1000 types have been identified (Yabuzaki 2017). The primary structure of carotenoids is formed by conjugated polyene chains and terminal groups. Cyclization, a terminal-group modification, contributes to the structural diversity of carotenoids. Lycopene, a noncyclic carotenoid, is converted to the dicyclic carotenoid β-carotene via cyclization of both terminal groups, which is abundantly found in various organisms, including plants. Conversely, some marine bacteria generate monocyclic carotenoids, such as myxol, via the cyclization of a single terminal group (Yokoyama and Miki 1995; Takatani et al. 2014, 2015).

Carotenoids exhibit numerous physiological functions, such as improving membrane stability, protecting against oxidative stress, and serving as accessory pigments during photosynthesis (Swapnil et al. 2021). Recent research has focused on the health benefits of carotenoids (Elvira-Torales et al. 2019). Deinoxanthin, a monocyclic carotenoid produced by extremophile microbes, such as *Deinococcus radiodurans*, exhibits strong scavenging activity against reactive oxygen species (ROS), such as hydrogen peroxide and singlet oxygen (Tian et al. 2007). Compared to the dicyclic carotenoids zeaxanthin and β-carotene, myxol and saproxanthin, other monocyclic carotenoids produced by marine bacteria, exhibit greater antioxidative and neuroprotective effects (Shindo et al. 2007). These findings indicate that the monocyclic structure of carotenoids exhibits various biological activities, including activities that provide health benefits. However, few monocyclic carotenoids have been identified, and thus, their biological activities are poorly understood.

Misawa et al. (1995) demonstrated that desired carotenoids can be obtained by expressing several biosynthetic genes isolated from marine bacteria in heterologous hosts, such as *Escherichia coli*, which should lead to the stable production of beneficial carotenoids and the generation of naturally rare carotenoids. Several unique genes involved in the biosynthesis of myxol, a representative monocyclic carotenoid, have been elucidated in marine bacteria; these genes include hydroxylase, desaturase, and lycopene cyclase (Rählert et al. 2009; Teramoto et al. 2003; Teramoto et al. 2004). However, only a few monocyclic carotenoid biosynthetic genes have been sequenced and functionally identified, and little is known about the monocyclic carotenoid biosynthesis system. Therefore, obtaining further knowledge about monocyclic carotenoid-producing bacteria and their carotenoid biosynthetic genes is important for future applications of monocyclic carotenoids.

In this study, we searched for marine bacteria that produce monocyclic carotenoids, and *Algoriphagus* sp. strain oki45 was isolated from the surface of unidentified seaweed collected in Okinawa, Japan. Structural analysis revealed that the strain oki45 produced monocyclic carotenoids flexixanthin and its hydroxylated derivative, which is a new compound. Furthermore, whole genome analysis of the strain oki45 and two related species found that several genes may be involved in the biosynthesis of those monocyclic carotenoids.

## Materials and methods

### Bacterial strains

Unidentified seaweeds collected in 2014 from Senaga-Jima Island (Okinawa, Japan) were soaked in artificial seawater and plated at 25 °C on Marine Agar 2216 (Difco). After several days of incubation, red colonies were selected and plated on fresh Marine Agar 2216. This process was repeated until only a single colony remained. The strain oki45 was deposited at the RIKEN BioResource Center, Japan Collection of Microorganisms (Tsukuba, Japan) under JCM 19877. *A. confluentis* NBRC 111222^ T^ (Park et al. 2016) and *A. taiwanensis* JCM 19755^ T^ (Shahina et al. 2014) were used for species identification and genome comparison. These strains were also cultured using Marine Agar 2216 at 25 °C.

### Carotenoid preparation

The strain oki45 was incubated for 1 week in 300 mL Marine Broth 2216 (Difco) in 500-mL Sakaguchi flasks at 140 rpm and 25 °C on a rotary shaker. The cell pellet was collected by centrifugation at 8000 rpm, and then the total lipid was extracted via the Folch method (Folch et al. 1957). The total lipid was separated on a preparative thin-layer chromatography (TLC) plate with silica gel 60 (Merck Millipore, Burlington, MA, USA) and *n*-hexane/ethyl acetate (40:60, v/v). Flexixanthin and 2-hydroxyflexixanthin, *Rf* 0.53 and 0.31, were scraped from silica gel and then eluted with acetone. Then, each carotenoid was purified using high-performance liquid chromatography (HPLC) with a Develosil ODS column (250 × 4.6 mm; Nomura Chemical Co., Inc., Aichi, Japan), eluting with methanol/acetonitrile/ethyl acetate (50:20:30, v/v/v) at a flow rate of 1.0 mL/min. Carotenoids were detected using a UV–Vis detector (Hitachi L-2400) at 470 nm.

### Spectroscopy

Absorption spectra were obtained using an HPLC LC-2050C 3D system (Shimadzu, Kyoto, Japan) equipped with a Mightysil Si 60 column (250 × 4.6 mm; Kanto Chemical Co., Inc., Tokyo, Japan). The column temperature and detection wavelength were set to 30 °C and 470 nm, respectively. The mobile phase comprised *n*-hexane/acetone (70:30, v/v) at a 1.0-mL/min flow rate. ^1^H and ^13^C nuclear magnetic resonance (NMR) spectra were acquired in CDCl_3_ using a UNITY INOVA-500 (Varian, USA) system and tetramethylsilane as an internal standard. The peak assignments of the homo and hetero two-dimensional NMR spectra were determined by comparing the data to those previously reported. The positive ion electrospray ionization (ESI)-mass spectrometry (MS) spectra data were obtained using an Acquity LC Xevo G2-S Q time-of-flight (TOF) MS spectrometer (Waters, USA). The ESI TOF MS spectra were acquired by scanning from *m/z* 100 to 1500 with a capillary voltage of 3.2 kV, a cone voltage of 40 eV, and a source temperature of 120 °C. Nitrogen was used as a nebulizing gas at a flow rate of 30 L/h. MS/MS spectra were measured using a quadrupole-TOF MS/MS instrument with argon and 30 V collision energy as the collision gas. UV–Vis spectra were recorded using a Hitachi U-2001 spectrometer (Hitachi Filed Navigator). Circular dichroism (CD) spectra data were obtained using a J-500C and J-1500 spectropolarimeter (Jasco, Tokyo, Japan) in ether at room temperature.

### Genome sequencing and downstream analyses

Genomic DNAs of strain oki45, *A. confluentis* NBRC 111222^ T^, and *A. taiwanensis* JCM 19755^ T^ were extracted and purified using the NucleoSpin Tissue kit (MACHEREY–NAGEL, Düren, Germany). Genome sequences were obtained using a MiSeq sequencer (Illumina, California, USA), and assembly was performed using Platanus B (Kajitani et al. 2014). The genome was further annotated using Rapid Annotation using Subsystem Technology (RAST) (Aziz et al. 2008). The genome data were deposited in DDBJ/EMBL/GenBank under the accession numbers BTPC01000001 to BTPC01000040, BTPD01000001 to BTPD01000050, and BTPE01000001 to BTPE01000051 for strains oki45, *A. confluentis* NBRC 111222^ T^, and *A. taiwanensis* JCM 19755^ T^, respectively.

### Molecular phylogenic analysis

**Fig. 1:**
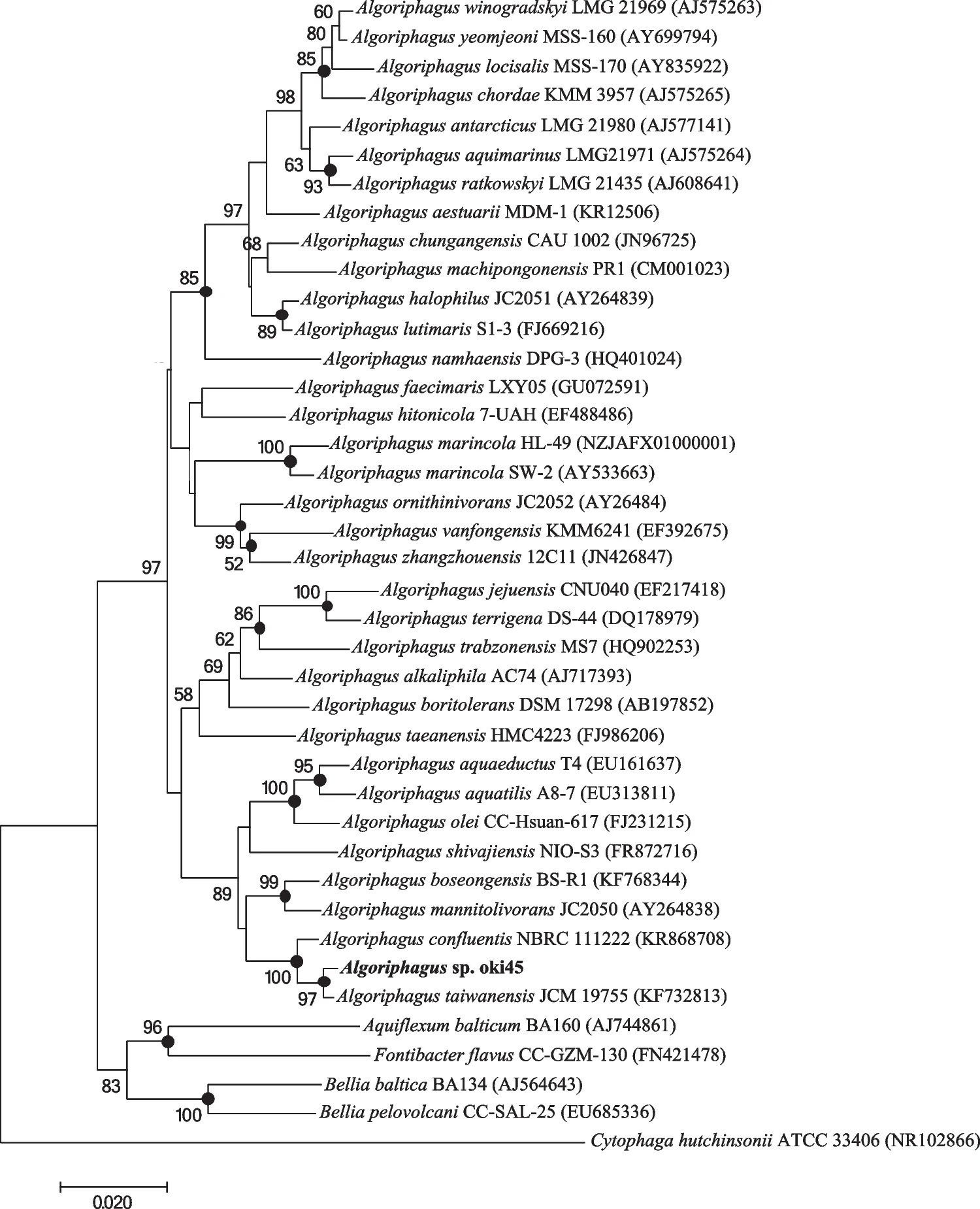
The molecular phylogenic tree based on 16S rRNA gene sequences of oki45 and *Algoriphagus* sp. This figure combines the results of three analyses, i.e., neighbor-joining, maximum-parsimony, and maximum-likelihood. The topology shown was obtained via neighbor-joining, and the percentage values are the results of a bootstrap analysis using 100 replications. Only bootstrap values > 50% are shown at branching points. Filled circles indicate the corresponding nodes, which were also recovered by maximum-likelihood and maximum-parsimony analyses

BLASTn (Mount 2007) at the National Center for Biotechnology Information (NCBI) site (Sayers et al. 2021) was used to search for and retrieve nucleotide sequences similar to the 16S ribosomal RNA (rRNA) gene sequence of strain oki45 from the nr database. The 16S rRNA gene nucleotide sequences of all *Algoriphagus*-type strains were retrieved from the GenBank/ENA/DDBJ database. The nucleotide sequence of the 16S rRNA gene of the strain oki45 was retrieved from the newly sequenced genome. The molecular phylogenetic tree depicted in Fig. 1 was reconstructed via the neighbor-joining method (Saitou and Nei 1987) after the nucleotide sequences were aligned using the MEGA version 7 program (Kumar et al. 2016). Maximum-likelihood and maximum-parsimony analyses were performed using MEGA7.

### Genome taxonomy

Average nucleotide identity (ANI) against the strain oki45 genome was calculated using OrthoANI (Lee et al. 2016). The Genome-to-Genome Distance Calculator (GGDC2) (Meier-Kolthoff et al. 2013) was used to calculate the in silico DDH value.

### Prediction of carotenoid biosynthetic genes

Sequence homology searches based on amino acid sequences were conducted to identify the carotenoid biosynthesis genes. The genes used for carotenoid biosynthesis were searched with reference to the biosynthetic system estimated by Tao et al. (2006). Genome sequences of known carotenoid biosynthetic genes were obtained from NCBI, and homology searches were performed using known carotenoid biosynthesis-related gene sequences on in silico MolecularCloning (IMC) (In silico Biology, Yokohama, Japan). We also used the Expert Protein Analysis System (ExPASy) (Gasteiger et al. 2003), BLAST, and Pfam (Finn et al. 2016) to estimate gene regions associated with protein classification and gene function. The NCBI Reference Sequence Database (RefSeq) (O’Leary et al. 2016) was used to determine the extent to which *Algoriphagus* sp. conserved 2-hydroxyflexixanthin biosynthesis genes.

## Results

### 16S rRNA gene phylogeny

The strain oki45 isolated from the surface of an unidentified seaweed formed a red water-insoluble-pigmented colony on Marine Agar 2216. A molecular phylogenetic analysis based on the 16S rRNA gene nucleotide sequence revealed that strain oki45 belonged to the genus *Algoriphagus* and shared 99.1% sequence similarity with *A. confluentis* NBRC 111222^ T^ and *A. taiwanensis* JCM 19755^ T^ (Fig. 1). The similarity to other *Algoriphagus* species sequences was less than 96.5%. *A. confluentis* NBRC 111222^ T^ and *A. taiwanensis* JCM 19755^ T^ produce red water-insoluble pigments, which have never been characterized.

### Structure determination of carotenoids produced by *Algoriphagus* sp. strain oki45

**Fig. 2:**
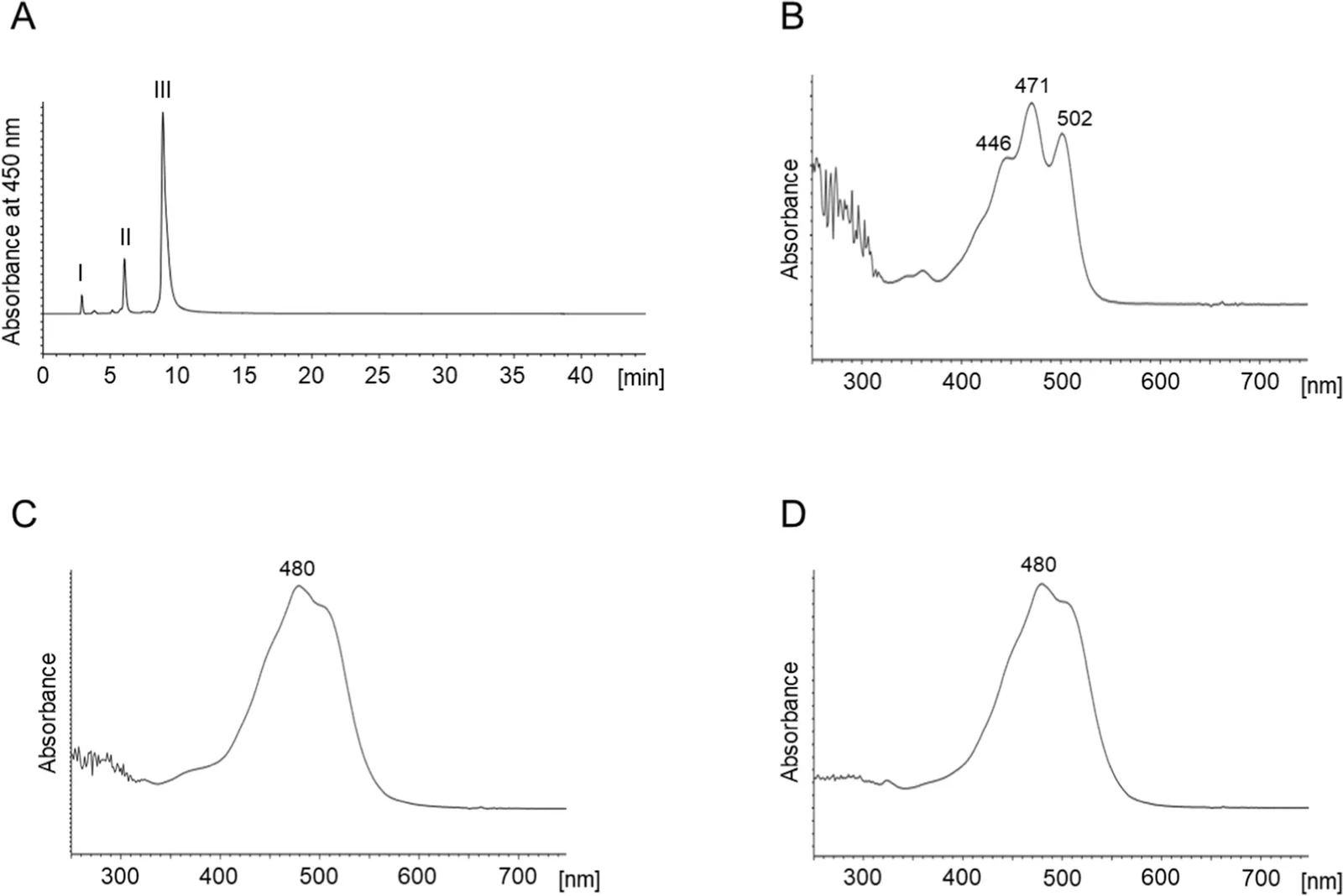
High-performance liquid chromatography profiles of the total lipid extracted from the strain oki45. **A** Chromatogram recorded at 470 nm. (**B**), (**C**), and (**D**) are the absorption spectra of peaks I, II, and III, respectively, depicted in (**A**)

To elucidate the molecular structure of carotenoids produced by strain oki45, total lipids were extracted and then analyzed using HPLC equipped with a silica gel column (Fig. 2). At 450 nm, the total lipids of the strain oki45 exhibited three major peaks (Fig. 2A). The minor peak I (2.92 min) corresponded to the authentic lycopene standard regarding the retention time and absorption spectrum (Figs. 2B and S1). Peaks II (6.08 min) and III (8.92 min) exhibited characteristic absorption spectra (Fig. 2C, D), indicating the presence of conjugated keto groups. To obtain detailed structural information, peaks II and III were purified via TLC and C18-HPLC. Then, high-resolution MS, NMR, and CD spectroscopy data were recorded.

**Fig. 3:**
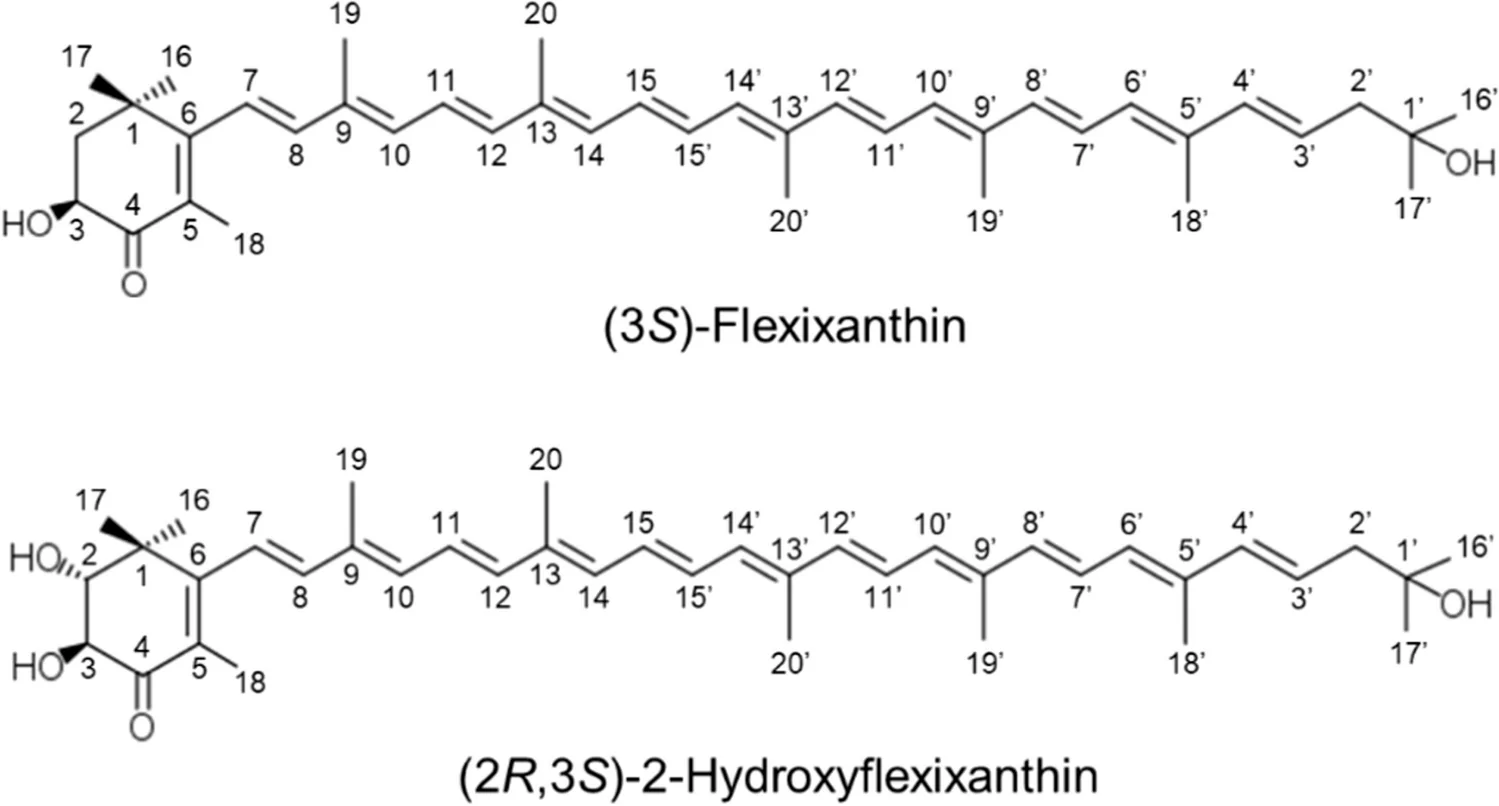
The structures of (3*S*)-flexixanthin and (2*R*,3*S*)-2-hydroxyflexixanthin produced by the strain oki45

Peak II showed a protonated molecule at *m/z* 583.4112 (calcd for C_40_H_55_O_3_, 583.4105) in ESI TOF MS. ESI TOF MS/MS showed characteristic product ions of [MH-H_2_O]^+^ (*m/z* 565.4016), [MH-2H_2_O]^+^ (*m/z* 547.3928), and [MH-74]^+^ (*m/z* 509.3411), indicating a monocyclic terminal group (Fig. S2). The ^1^H NMR and CD spectroscopic data (Table 1 and Figs. S3–S6) were identical to those reported for (3*S*)-flexixanthin ((3*S*)-3,1′-dihydroxy-3′,4′-didehydro-1′,2′-dihydro-β,ψ-caroten-4-one) (Aasen and Jensen 1966; Coman and Weedon 1975; Andrewes et al. 1984). Therefore, this pigment was identified as (3*S*)-flexixanthin (Fig. 3).

**Table 1:** Nuclear magnetic resonance data of peaks II and III

	Peak II	Peak III	
Position	*δ*_H_ (*J* in Hz)	*δ*_C_	δ_H_ (*J* in Hz)
1		41.8	
2	1.81, dd (13.5, 13.5)	77.5	3.53, d (11.5)
2	2.15, dd (13.5, 5.5)		
3	4.32, ddd (14.5, 6)	74.1	4.17, dd (11.5, 1.85)
4		198.2	
5		126.9	
6		162.1	
7	6.22, d (16)	122.6	6.25, d (16)
8	6.43, d (16)	142.8	6.45, d (16)
9		135.0	
10	6.31, d (11)	135.5	6.31, d (11)
11	6.66, dd (15, 11)	124.2	6.66, dd (15, 11)
12	6.45, d (15)	140.0	6.45, d (15)
13		136.3	
14	6.25, overlapped	134.1	6.31, d (11)
15	6.64, overlapped	130.0	~ 6.65, m
16	1.33, s	20.1	1.27, s
17	1.21, s	25.7	1.30, s
18	1.95, s	14.0	1.94, s
19	2.01, s	12.8	2.00, s
20	1.99, s	12.8	1.99, s
1′		72.7	
2′	2.32, d (7.5)	56.4	2.31, d (8)
3′	5.76, dt (15, 7.5)	124.5	5.77, dt (15, 8)
4′	6.22, d (15)	138.8	6.22, d (15)
5′		137.2	
6′	6.14, d (11)	131.2	6.14, d (11)
7′	6.61, dd (15, 11)	124.7	6.61, dd (16, 11)
8′	6.44, d (15)	137.8/137.9	6.36, d (16)
9′		136.2	
10′	6.37, d (11.5)	132.7	6.25, d (11)
11′	6.64, overlapped	125.4	6.64, dd (15, 11)
12′	6.38, d (15)	137.8/137.9	6.39, d (15)
13′		136.3	
14′	6.25, overlapped	132.8	6.28, d (11)
15′	6.64, overlapped	131.0	~ 6.65, m
16′	1.24, s	29.2	1.24, s
17′	1.24, s	29.2	1.24, s
18′	1.94, s	13.0	1.94, s
19′	1.99, s	12.9	1.99, s
20′	1.99, s	12.6	1.99, s
3-OH	3.69, s		3.68, d (2)

*s* singlet, *d* doublet, *dd* doublet of doublets, *ddd* a doublet of doublets of doublets, *m* multiplet, *dt* doublet of triplets

Peak III exhibited UV–Vis absorption maxima at 476 nm and a molecular ion at *m/z* 598.4012 (calcd for C_40_H_54_O_4_, 598.4022). ESI TOF MS/MS showed characteristic product ions of [M-92]^+^ (*m/z* 506.3360) and [M-58]^+^ (*m/z* 540.3564), indicating a monocyclic terminal group (Fig. S7). The ^1^H and ^13^C NMR data for this compound are listed in Table 1 (Figs. S8–S16, S22, and S23). These NMR data were assigned by correlation spectroscopy (COSY), nuclear Overhauser effect spectroscopy (NOESY), heteronuclear single-quantum correlation spectroscopy (HSQC), and heteronuclear multiple-bond correlation spectroscopy (HMBC) and compared to previously published NMR data of carotenoids (Englert 1995; Yokoyama et al. 1996). Based on the UV–Vis and MS spectroscopic data, this carotenoid was assumed to be a hydroxyl derivative of flexixanthin. The COSY spectrum revealed a 11.5-Hz coupling between oxymetin protons at *δ* 3.53 and 4.17. This indicated that the protons of oxymetin were in the vicinal position and exhibited a *trans* configuration (Englert 1995; Yokoyama et al. 1996). Furthermore, the ^1^H and ^13^C NMR data of C-1 to C-6, including CH_3_ groups at positions 16, 17, and 18, of this compound were identical to those of 2-hydroxyastaxanthin (Yokoyama et al. 1996). Therefore, the position of the other hydroxyl group at the C-2 position of flexixanthin was determined. The CD spectrum of this compound (Fig. S17) showed [(EPA) nm (Δ*ε*) 240 (− 0.5), 245 (0), 270 (+ 2.0), 275 (0), 310 (− 11.8), 329 (0), 360 (+ 1.2)]) wavelengths similar to those described for (3*S*)-flexixanthin (Andrewes et al. 1984). It was reported that the chirality of the hydroxy group at C-3 primarily contributed to the shape of the CD spectrum of the 2,3-dihydroxy β-end group (Bucheker and Noack 1995). Consequently, it was found that this compound has the same chirality as (3*S*)-flexixanthin. The chirality of the hydroxyl group at the C-2 position was determined using NMR data pertaining to the relative configuration of the C-3 position. Thus, the compound was identified as ((2*R*,3*S*)-2,3,1′-trihydroxy-3′,4′-didehydro-1′,2′-dihydro-β,ψ-caroten-4-one) and designated as (2*R*,3*S*)-2-hydroxyflexixanthin (Fig. 3). Furthermore, *A. confluentis* NBRC 111222^ T^ and *A. taiwanensis* JCM 19755^ T^ also produced flexixanthin and 2-hydroxyflexixanthin (Fig. S18).

### Prediction of carotenoid biosynthetic genes

To search for genes involved in the biosynthesis of flexixanthin and 2-hydroxyflexixanthin, we compared genome sequences of the strains oki45, *A. confluentis* NBRC 111222^ T^, and *A. taiwanensis* JCM 19755^ T^. The genome sequence of strain oki45 was estimated to be 4,617,600 bp with 40 contigs. The draft genome sequences of *A. confluentis* NBRC 111222^ T^ and *A. taiwanensis* JCM 19755^ T^ were 5,014,366 bp with 54 contigs and 4,563,290 bp with 51 contigs, respectively. The GC contents of oki45, *A. confluentis* NBRC 111222^ T^ and *A. taiwanensis* JCM 19755^ T^ were 43.8, 44.1, and 43.9%, respectively, and the putative protein coding regions (CDSs) were 4347, 4802, and 4315, respectively. In addition, 1957 CDSs, including carotenoid biosynthesis genes, were shared by oki45, *A. confluentis* NBRC 111222^ T^, and *A. taiwanensis* JCM 19755^ T^ (Fig. S19).

**Fig. 4:**
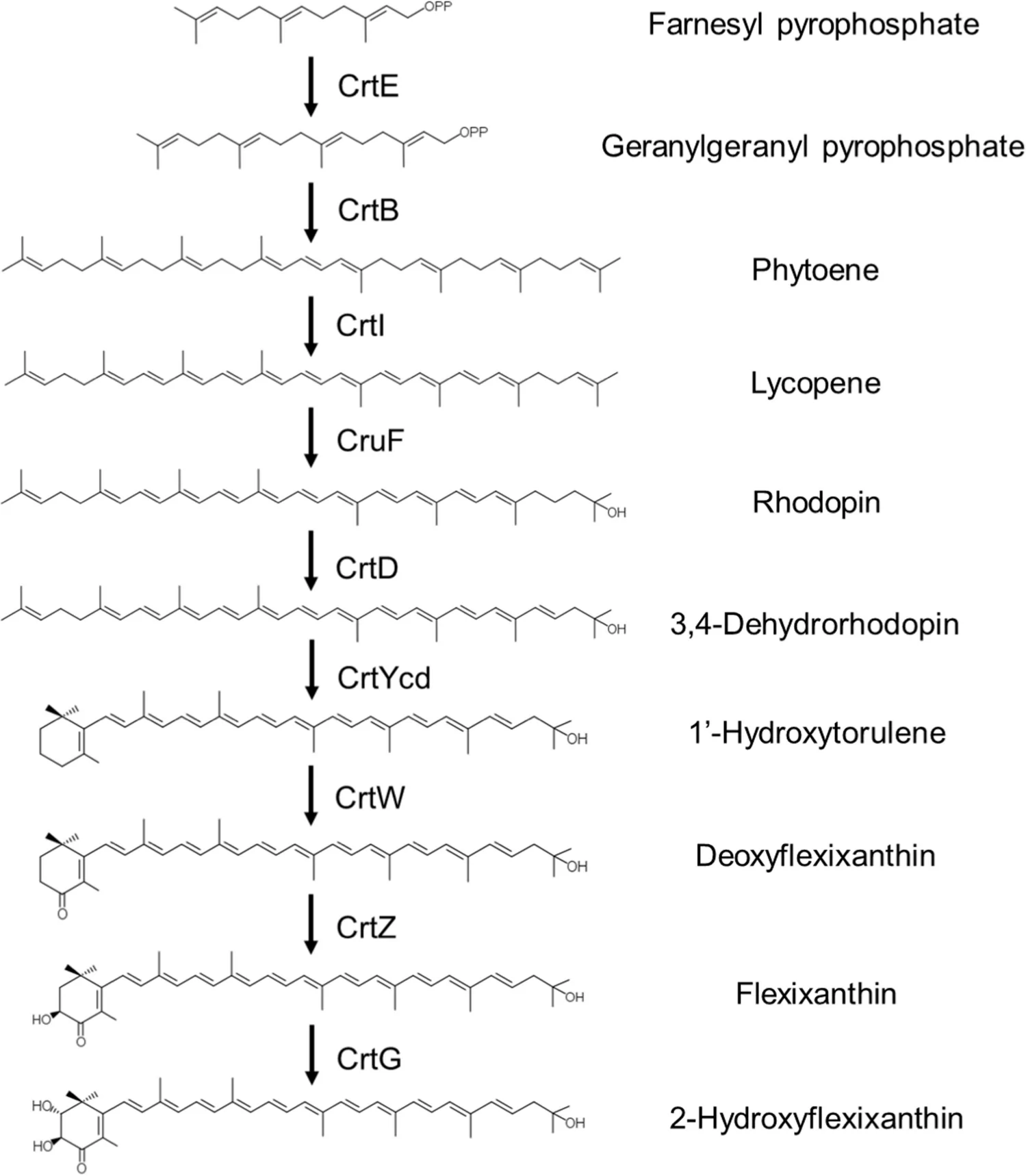
Proposed carotenoid biosynthetic pathway in the strain oki45. CrtE, geranylgeranyl pyrophosphate synthase; CrtB, phytoene synthase; CrtI, phytoene dehydrogenase; CruF, 1,2-hydratase; CrtD, desaturase; CrtYcd, lycopene cyclase; CrtW, β-ionone ring ketolase; CrtZ, β-ionone ring hydroxylase; CrtG, 2,2ʹ-β-hydroxylase

Eight carotenoid biosynthetic genes (*crtE*, *crtB*, *crtI*, *cruF*, *crtD*, *crtYcd*, *crtW*, and *crtZ*) were predicted in the strain oki45 genome through a homology search (Table 2), which were presumed to be necessary for flexixanthin biosynthesis (Fig. 4). Interestingly, a gene homologous to *crtG* encoding 2,2ʹ-β-hydroxylase was found in the genome of strain oki45 (Table 2). These nine carotenoid biosynthetic genes were also present in the genomes of *A. confluentis* NBRC 111222^ T^ and *A. taiwanensis* JCM 19755^ T^ (Fig. S20).

**Table 2:** Proposed carotenoid biosynthetic genes in *Algoriphagus* sp. strain oki45

Name	Amino acid length	Description	Amino acid identity (%)	Organism	GenBank accession number
*crtE*	328	Geranylgeranyl pyrophosphate synthase	68.9	*Cecembia lonarensis* LW9	EK47446
*crtB*	280	Phytoene synthase	77.4	*Algoriphagus* sp. strain KK10202C	DQ286432
*crtI*	494	Phytoene dehydrogenase	83.3	*Algoriphagus* sp. strain KK10202C	DQ286432
*cruF*	221	1,2-Hydratase	32.1	*Haloarcula japonica* TR-1	LC008544
*crtD*	493	Desaturase	54.5	*Flavobacterium* sp. P99-3	AB97813
*crtYcd*	246	Lycopene cyclase	74.4	*Algoriphagus* sp. strain KK10202C	DQ286432
*crtW*	234	β-Ionone ring ketolase	71.6	*Algoriphagus* sp. strain KK10202C	DQ286432
*crtZ*	148	β-Ionone ring hydroxylase	42.9	*Flavobacterium* sp. P99-3	AB97813
*crtG*	269	2,2ʹ-β-Hydroxylase	40.4	*Brevundimonas* sp. SD212	AB181388

## Discussion

Recently, carotenoids have attracted attention due to their health benefits (Elvira-Torales et al. 2019). Monocyclic structure of carotenoids can be associated with several biological activities, such as antioxidative and neuroprotective effects (Shindo et al. 2007) and neurogenesis activity (Kim et al. 2016). However, only a small number of monocyclic carotenoids have been identified, and information about their producers is limited. Some marine bacteria produce monocyclic carotenoids, and their biosynthetic genes have been found (Rählert et al. 2009; Teramoto et al. 2003; Teramoto et al. 2004). For future applications of monocyclic carotenoids, such as bioactive components and alternative production in heterologous hosts, it is necessary to accumulate knowledge on monocyclic carotenoid-producing bacteria and their biosynthetic genes.

The genus *Algoriphagus* in the family *Cyclobacteriaceae* was first proposed by Bowman et al. (2003). It has been isolated from various sources, such as Antarctic sea ice, seawater, and sediment. Although the bacteria are known to produce an orange-to-red color, their pigment composition has not been completely clarified. Herein, strain oki45 was isolated from the surface of a seaweed collected in Okinawa, and 16S rRNA phylogenetic analysis revealed that the strain belonged to the genus *Algoriphagus*. The strain oki45 was closely related to *A. confluentis* NBRC 111222^ T^ and *A. taiwanensis* JCM 19755^ T^ with 99.1% nucleotide sequence similarity (Fig. 1). Further ANI calculations using newly sequenced genomes of oki45, *A. confluentis* NBRC 111222^ T^ and *A. taiwanensis* JCM 19755^ T^, revealed 85.4% and 88.5% ANI of oki45 against *A. confluentis* NBRC 111222^ T^ and *A. taiwanensis* JCM 19755^ T^, respectively, which is below the species threshold of 95% (Fig. S21). The in silico DDH similarity of strain oki45 against *A. confluentis* NBRC 111222^ T^ and *A. taiwanensis* JCM 19755^ T^ was 35.7% and 29.5%, respectively, which is also below the species boundary of 70% (Table S1). Therefore, it was suggested that the strain oki45 may be a new species of the genus *Algoriphagus*.

Structural analysis revealed that the strain oki45 produced both flexixanthin and its hydroxylated derivative, 2-hydroxyflexixanthin (Figs. 2 and 3). Deinoxanthin, a 2-hydroxyl monocyclic carotenoid, helps the bacteria grow in extreme environments by quenching intracellular ROS in radio-tolerant *D. radiodurans* (Tian et al. 2007). Interestingly, the C-2 hydroxy group of deinoxanthin was shown to play an essential role in ROS quenching (Zhou et al. 2015). Furthermore, 2-hydroxylation of canthaxanthin, although not a monocyclic carotenoid, enhanced its inhibitory effect on lipid peroxidation (Nishida et al. 2005). Therefore, 2-hydroxyflexixanthin, which possesses a hydroxy group at the C-2 position, may also exhibit antioxidant activity.

Tao et al. (2006) reported that *Algoriphagus* sp. strain KK10202C produces flexixanthin. Furthermore, they identified four genes involved in flexixanthin biosynthesis in the strain KK10202C, i.e., phytoene synthase (*crtB*), phytoene dehydrogenase (*crtI*), lycopene cyclase (*crtYcd*), and β-ionone ring ketolase (*crtW*), which were found in a cluster (Tao et al. 2006). However, the presence of other carotenogenic genes, such as geranylgeranyl pyrophosphate synthase (*crtE*), desaturase (*crtD*), 1,2-hydratase, and β-ionone ring hydroxylase (*crtZ*), was not clear. Although *crtC* might be involved in the conversion of lycopene to rhodopin as a 1,2-hydratase in the strain KK10202C (Tao et al. 2006), a *crtC* homolog was not found in the genome of strain oki45, *A. confluentis* NBRC 111222^ T^ or *A. taiwanensis* JCM 19755^ T^. A *cruF* gene, which is an ortholog of *crtC*, is responsible for the 1,2-hydratase of γ-carotene in the nonphotosynthetic bacteria *D. radiodurans* R1 and *D. geothermalis* DSM 11300 (Sun et al. 2009). Gene *cruF* is known to be evolutionarily distant from *crtC*. Since the *cruF* gene was involved in a cluster with *crtZ*, *crtI*, *crtB*, *crtYcd*, and *crtW* (Fig. S20), it was speculated that the *cruF* gene could associate with C-1,2 hydration in carotenoids produced by *Algoriphagus* bacteria.

Genes encoding 2,2ʹ-β-hydroxylase were found for the first time in the strain oki45, *A. confluentis* NBRC 111222^ T^, and *A. taiwanensis* JCM 19755^ T^ (Fig. S20), and these *Algoriphagus* strains can biosynthesize 2-hydroxyflexixanthin (Fig. S18). Interestingly, the genes were homologous to *crtG* in *Brevundimonas* sp. SD212, which has been confirmed to catalyze C-2(2ʹ)-hydroxylation of the β-ionone ring (Nishida et al. 2005). Furthermore, the *crtG* gene product of *Thermosynechococcus elongatus* strain BP-1 catalyzes the 2-hydoxylation of myxol glycosides (Iwai et al. 2008). Therefore, *Algoriphagus*’s *crtG* genes may be involved in 2-hydroxyflexixanthin biosynthesis (Fig. 4). To estimate gene function, in vitro assays using isolated enzymes and in vivo assays using gene knockout systems or gene expression systems in heterologous hosts are performed. Most of the carotenoid biosynthetic genes encode membrane-associated enzymes, making it difficult to isolate them while retaining enzymatic activity. Furthermore, according to our knowledge, no knockout system for carotenoid biosynthesis genes in *Algoriphagus* bacteria has been established. On the other hand, carotenoid production in *E. coli* is useful in that there is no need to isolate the enzyme. However, in order to produce the desired carotenoid in *E. coli*, it is necessary to introduce a large number of genes and to stably express all of them. Even the production of the precursor flexixanthin in *E. coli* has not yet been established. Further studies of these *crtG* genes, including functional analysis using heterologous hosts, are needed in the future.

In conclusion, we isolated *Algoriphagus* sp. strain oki45 from the surface of unidentified seaweed collected in Okinawa, Japan. Two red pigments produced by the strain oki45, (3*S*)-flexixanthin and (2*R*,3*S*)-2-hydroxyflexixanthin, were identified as rare and novel monocyclic carotenoids. Two *Algoriphagus* strains that were related to the strain oki45 also synthesized both monocyclic carotenoids. Furthermore, whole genome analysis found eight genes (*crtE*, *crtB*, *crtI*, *cruF*, *crtD*, *crtYcd*, *crtW*, and *crtZ*) for flexixanthin biosynthesis. In addition, a gene with homology to 2,2ʹ-β-hydroxylase-encoding *crtG* was found in the strains oki45, *A. confluentis* NBRC 111222^ T^, and *A. taiwanensis* JCM 19755^ T^, suggesting that the gene is involved in 2-hydroxyflexixanthin biosynthesis. These findings expand our knowledge of monocyclic carotenoid biosynthesis in *Algoriphagus* bacteria.

## Supplementary Information

Below is the link to the electronic supplementary material.

Supplementary file1 (PDF 1695 KB)

## Data Availability

All data generated or analyzed during this study are included in this published article and its supplementary information file.
