# Supplementary material for: Identification of a novel monocyclic carotenoid and prediction of its biosynthetic genes in Algoriphagus sp. oki45
Source: Appl Microbiol Biotechnol. 2024 Jan 11;108:102. doi: 10.1007/s00253-023-12995-2 (PMC10784355; doi:10.1007/s00253-023-12995-2)
Supplement: Supplementary file 1 [file 253_2023_12995_MOESM1_ESM.pdf]

Supplementary Information

Applied Microbiology and Biotechnology

Identification of a novel monocyclic carotenoid and prediction of its biosynthetic genes in *Algoriphagus* sp. oki45

Naoki Takatani[1], Takashi Maoka[2], Tomoo Sawabe[1], Fumiaki Beppu[1], Masashi Hosokawa[1,*]

[1] Faculty of Fisheries Sciences, Hokkaido University, 3-1-1 Minato, Hakodate, Hokkaido 041-8611, Japan

[2] Research Institute for Production Development, 15 Shimogamo-morimoto-cho, Sakyo-ku, Kyoto 606-0805, Japan

*Corresponding author

E-mail: hoso@fish.hokudai.ac.jp

Tel & Fax: +81-138-40-5530

Contents

Supplementary Figures

Fig. S1 Comparison of HPLC chromatogram and absorption spectrum between lycopene standard and Peak I in total lipids extracted from the strain oki45 ··························· 4

Fig. S2 TOF MS/MS spectrum of purified Peak II ··························· 5

Fig. S3 ^{1}H-NMR of purified Peak II ··························· 6

Fig. S4 COSY spectrum of purified Peak II ··························· 7

Fig. S5 NOESY spectrum of purified Peak II ··························· 8

Fig. S6 CD spectrum of purified Peak II ··························· 9

Fig. S7 TOF MS/MS spectrum of purified Peak III ··························· 10

Fig. S8 ^{1}H-NMR of purified Peak III ··························· 11

Fig. S9 ^{13}C-NMR of purified Peak III ··························· 12

Fig. S10 COSY spectrum of purified Peak III ··························· 13

Fig. S11 NOESY spectrum of purified Peak III ··························· 14

Fig. S12 HSQC spectrum of purified Peak III ··························· 15

Fig. S13 Enlarged view of HSQC spectrum in Figure S12 ··························· 16

Fig. S14 HMBC spectrum of purified Peak III ··························· 17

Fig. S15 Enlarged view 1 of HMBC spectrum in Figure S14 ··························· 18

Fig. S16 Enlarged view 2 of HMBC spectrum in Figure S14 ··························· 19

Fig. S17 CD spectrum of purified Peak III ··························· 20

Fig. S18 TLC analysis of *Algoriphagus confluentis* NBRC 111222^{T} and *Algoriphagus taiwanensis* JCM 19755^{T} ··························· 21

Fig. S19 Venn-Diagram comparing the genomic content of three *Algoriphagus* ··························· 22

Fig. S20 Putative carotenoid biosynthetic genes in strain oki45, *A. confluentis* NBRC 111222^T, *A. taiwanensis* JCM 19755^T ··· 23

Fig. S21 Comparison of ANI between 10 *Algoriphagus* ··· 24

Fig. S22 1D NOE spectrum of purified Peak III (1) ··· 25

Fig. S23 1D NOE spectrum of purified Peak III (2) ··· 26

Supplementary Table

Table S1 Calculation results using GGDC for three strains of oki45, *A. confluentis* NBRC 111222^T, *A. taiwanensis* JCM 19755^T ··· 27

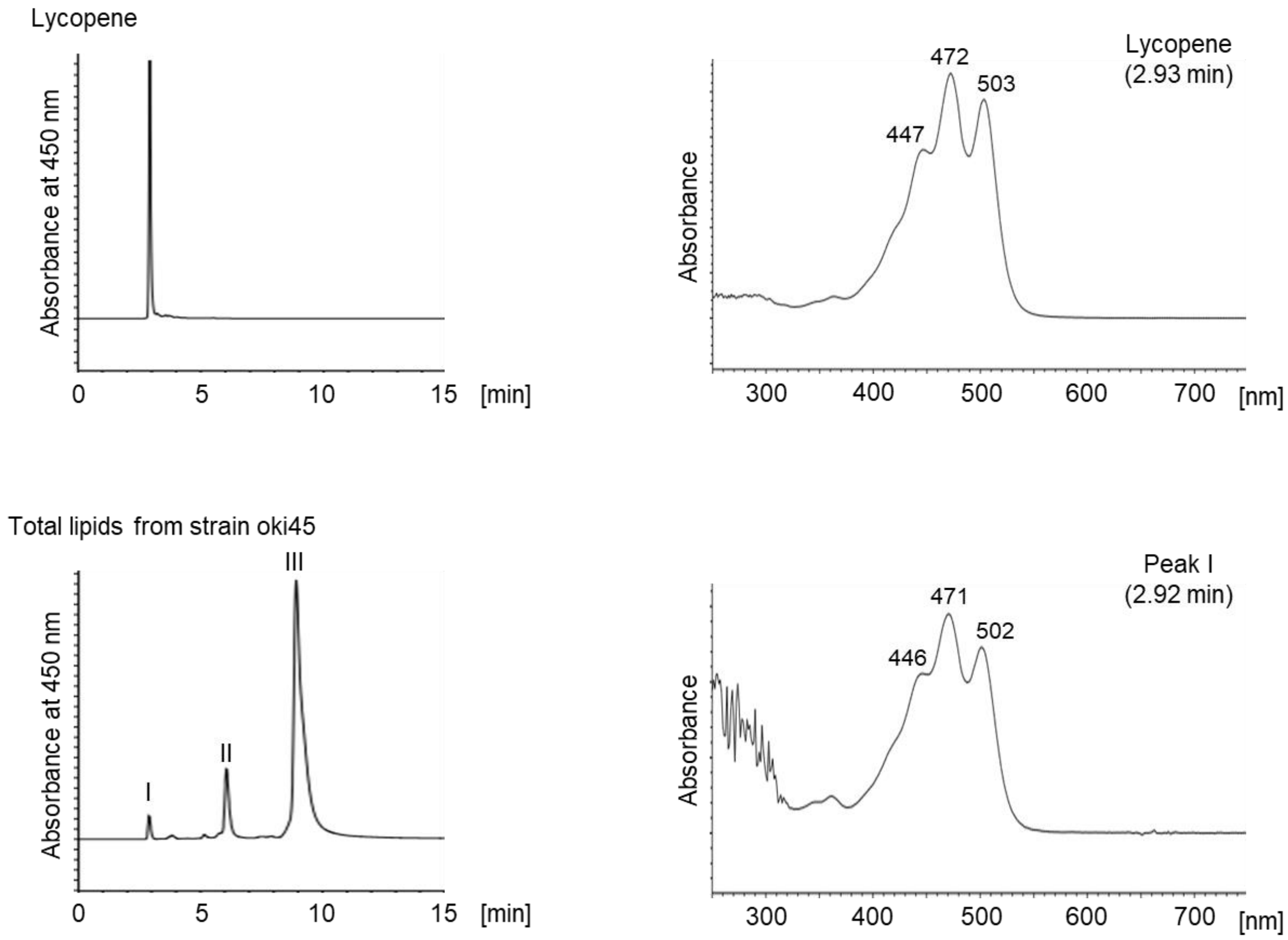

Fig. S1 Comparison of HPLC chromatogram and absorption spectrum between lycopene standard (upper panels) and Peak I in total lipids extracted from the strain oki45 (lower panels).

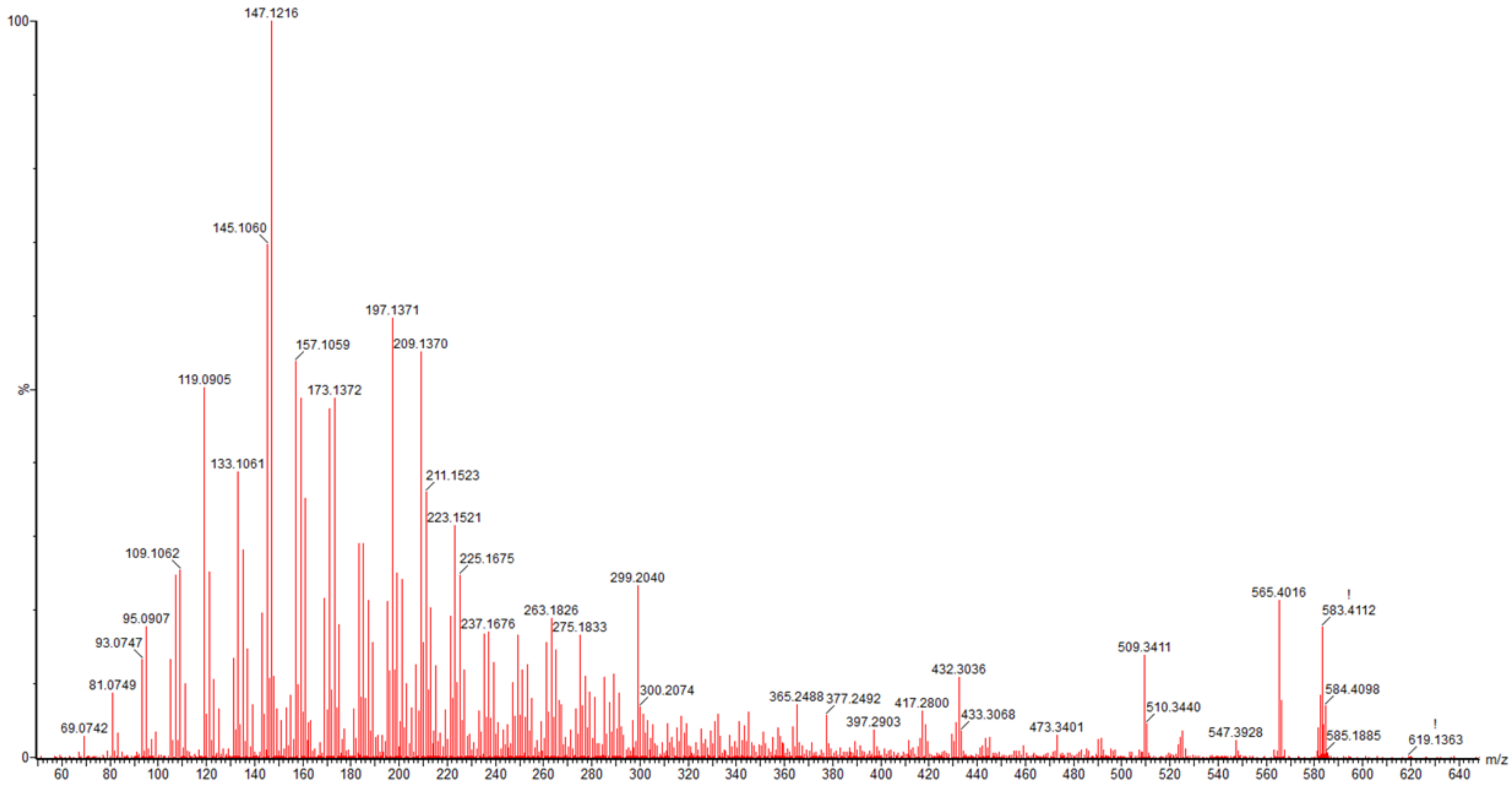

Fig. S2 TOF MS/MS spectrum of purified Peak II

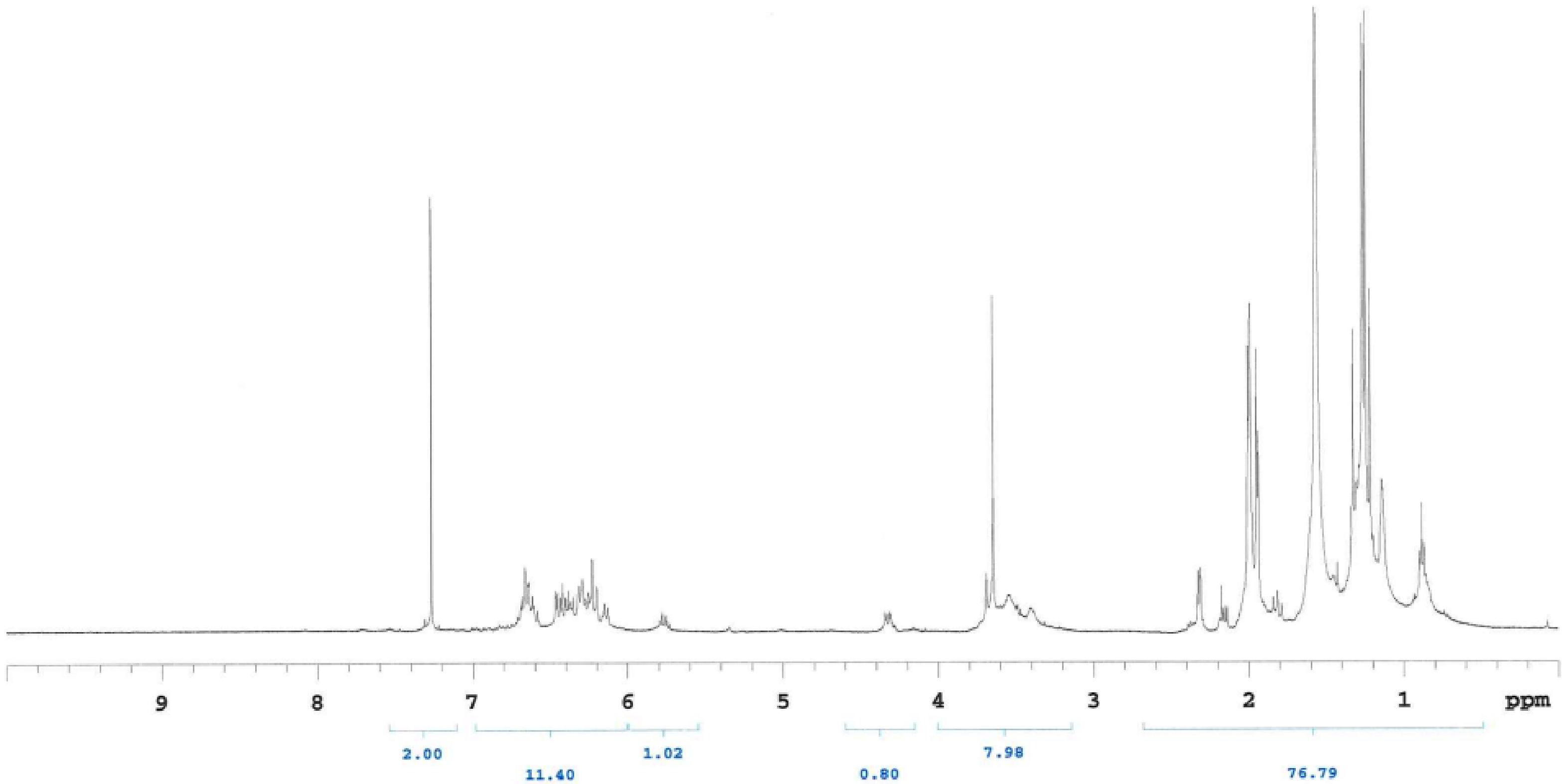

Fig. S3 ^{1}H-NMR of purified Peak II

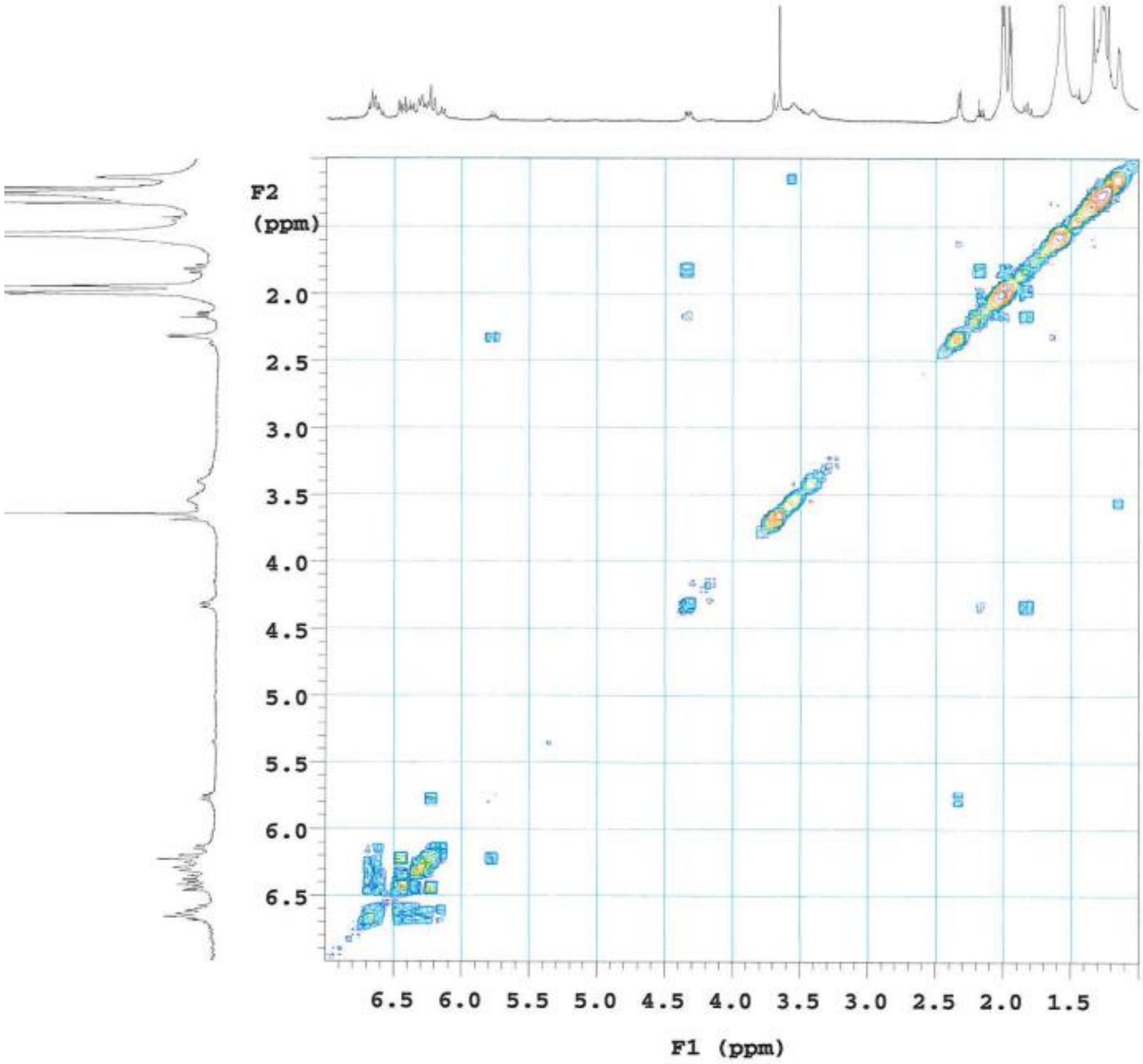

Fig. S4 COSY spectrum of purified Peak II

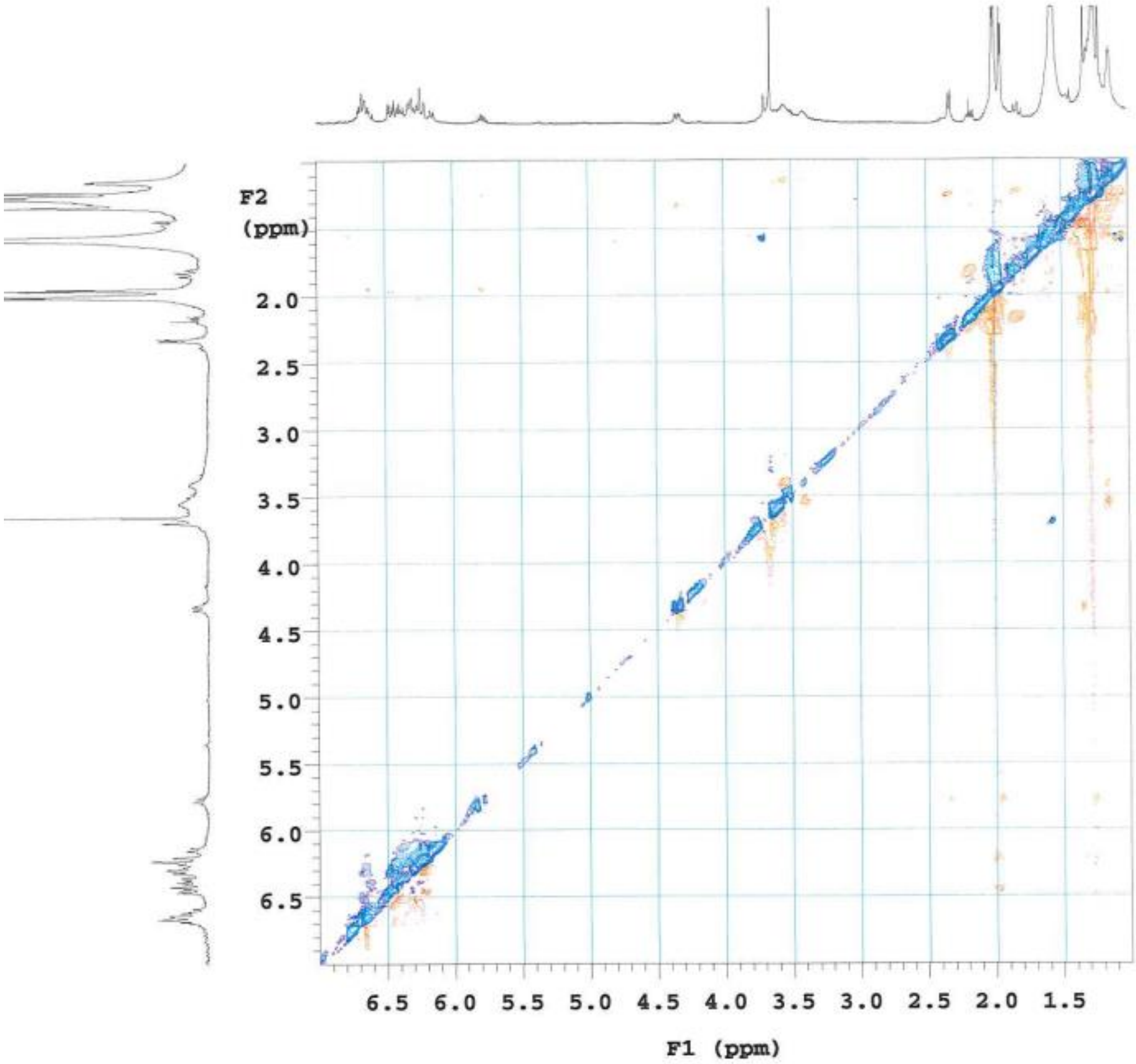

Fig. S5 NOESY spectrum of purified Peak II

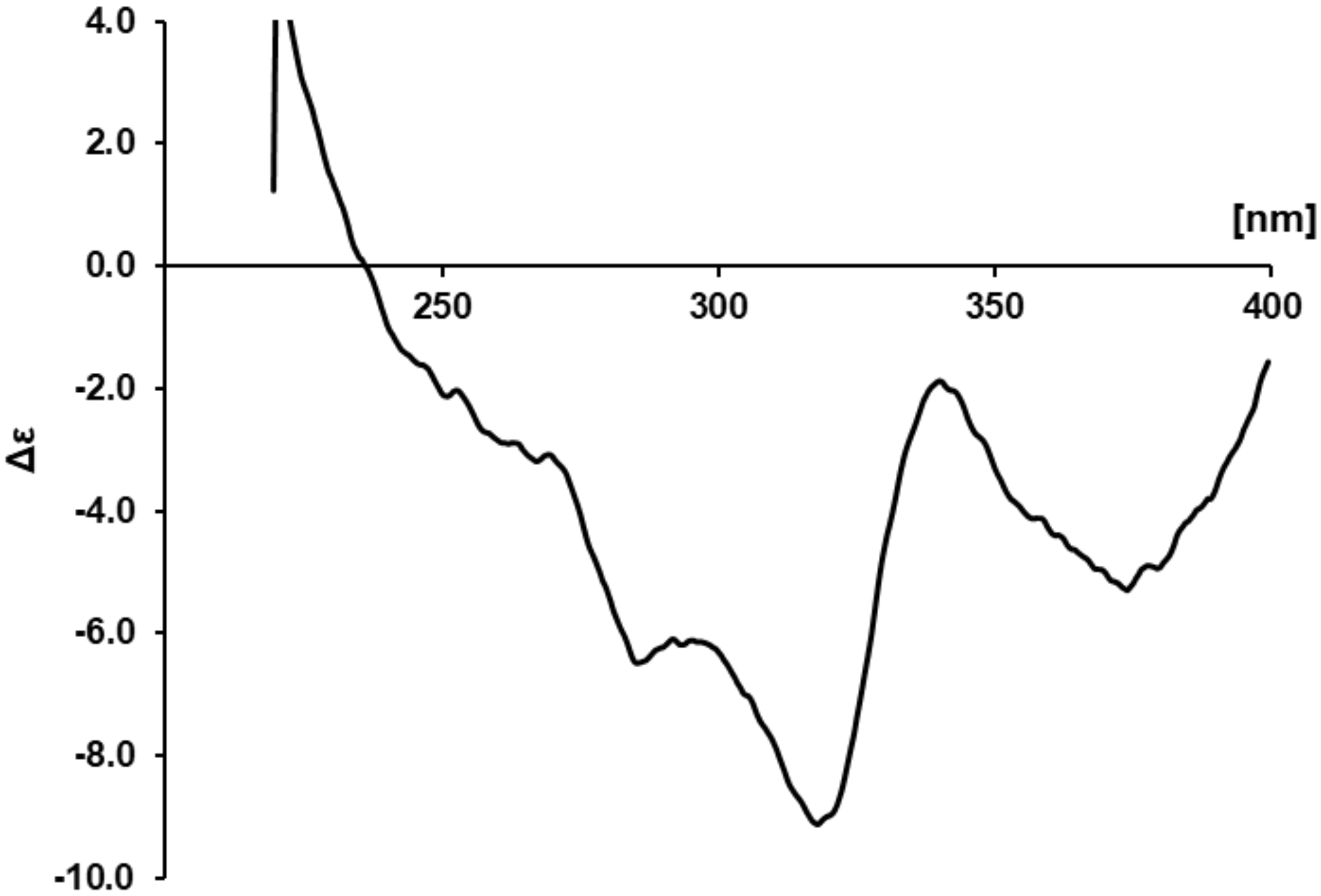

Fig. S6 CD spectrum of purified Peak II

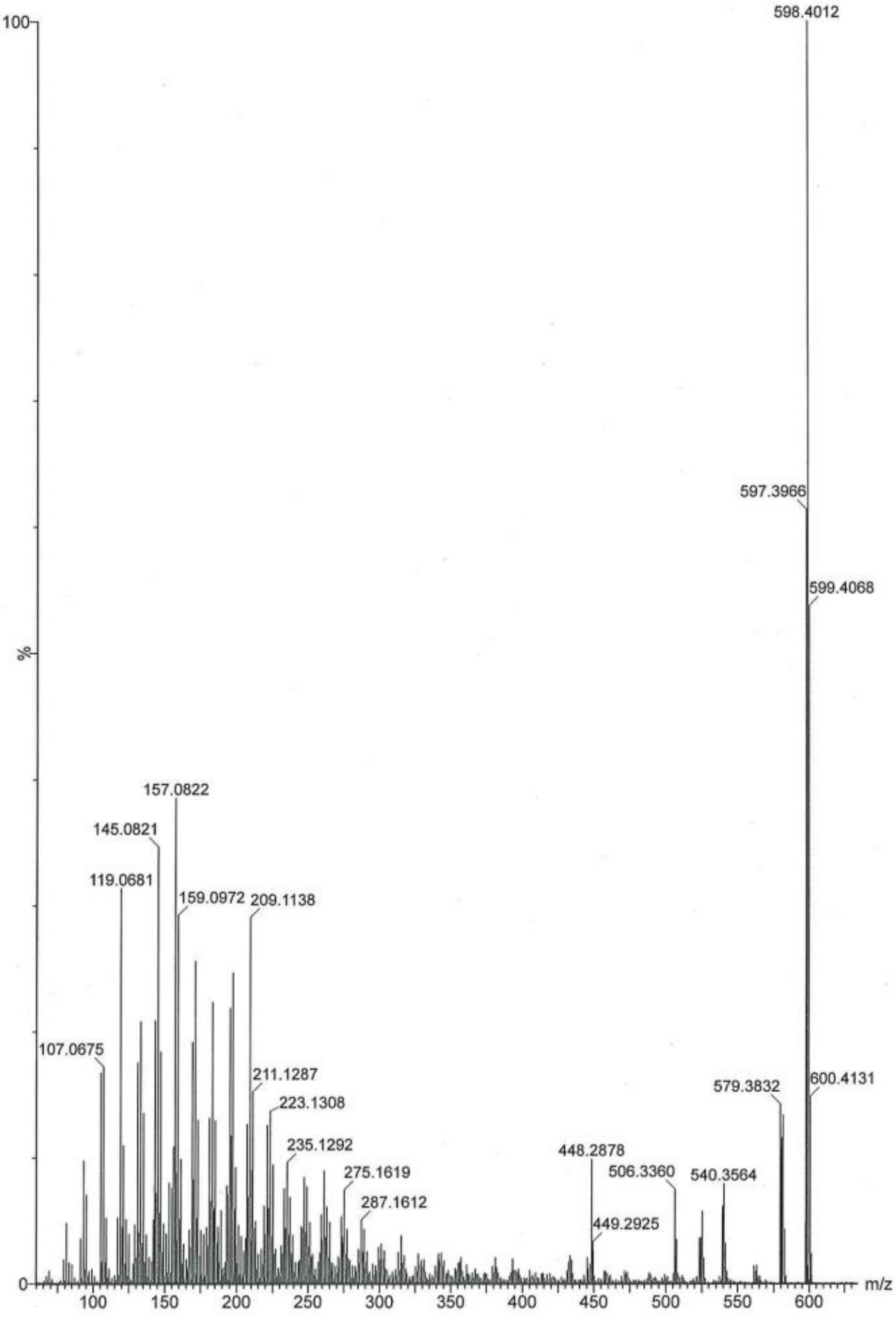

Fig. S7 TOF MS/MS spectrum of purified Peak III

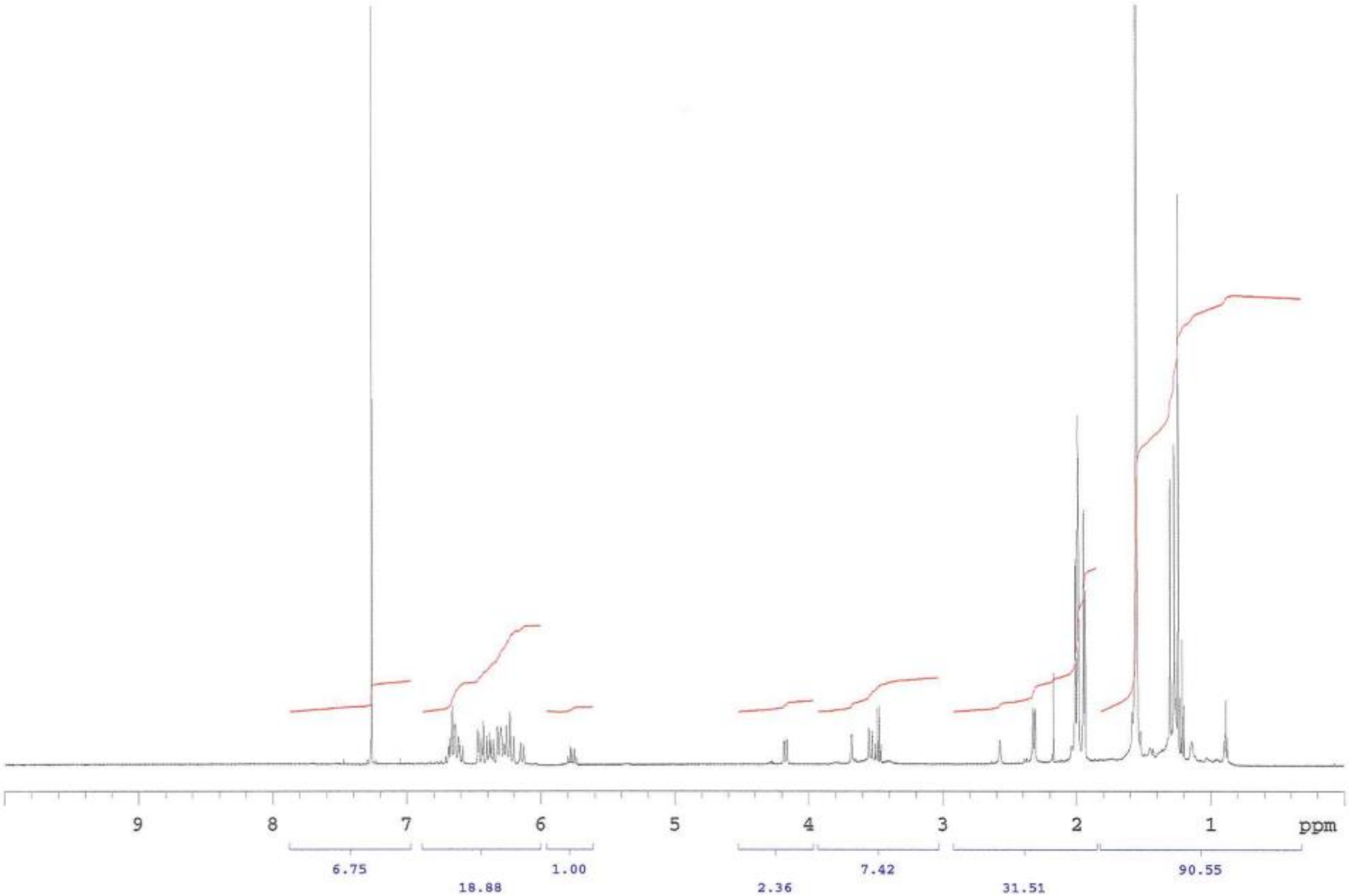

Fig. S8 ^{1}H-NMR of purified Peak III

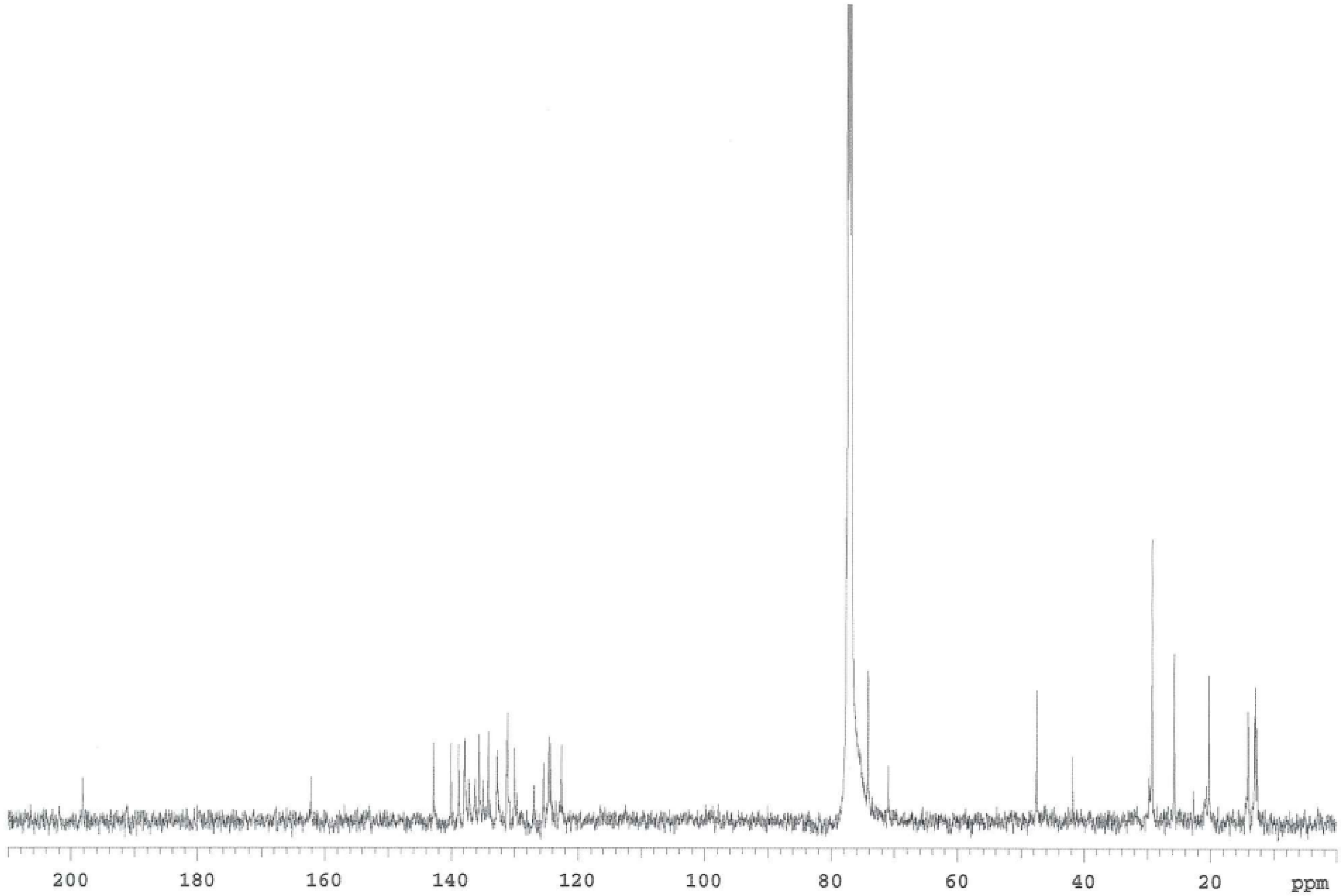

Fig. S9 ^{13}C-NMR of purified Peak III

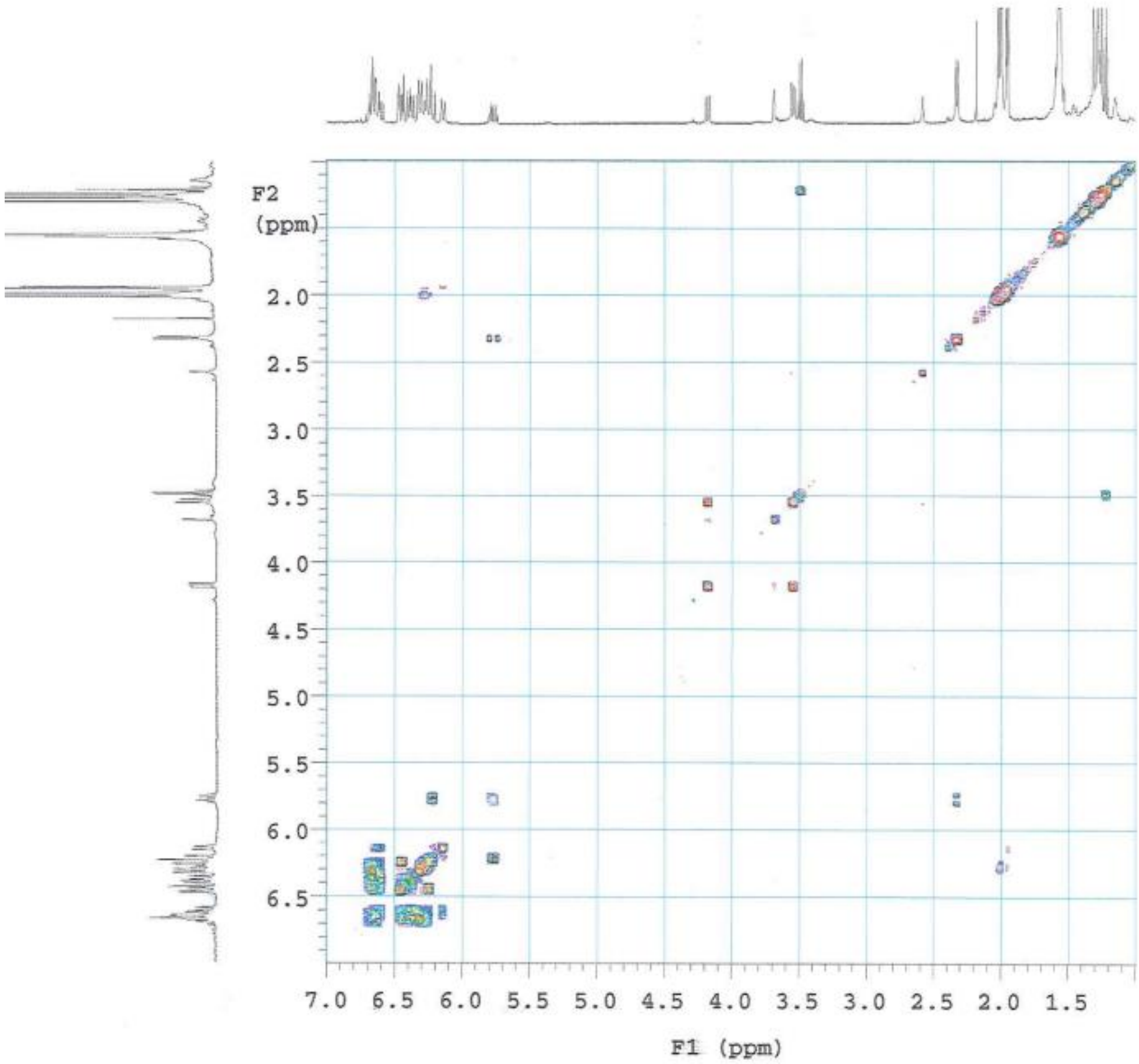

Fig. S10 COSY spectrum of purified Peak III

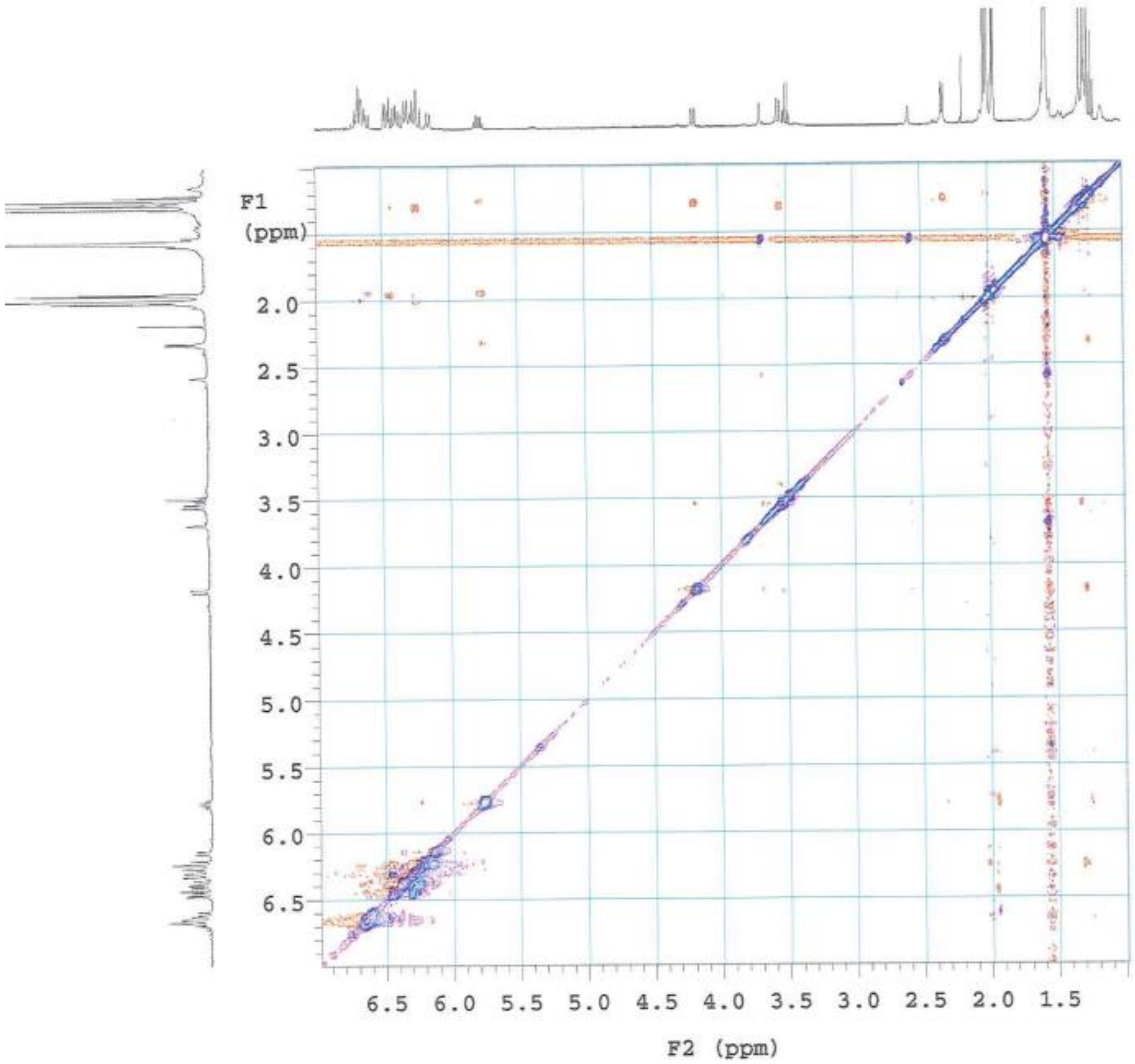

Fig. S11 NOESY spectrum of purified Peak III

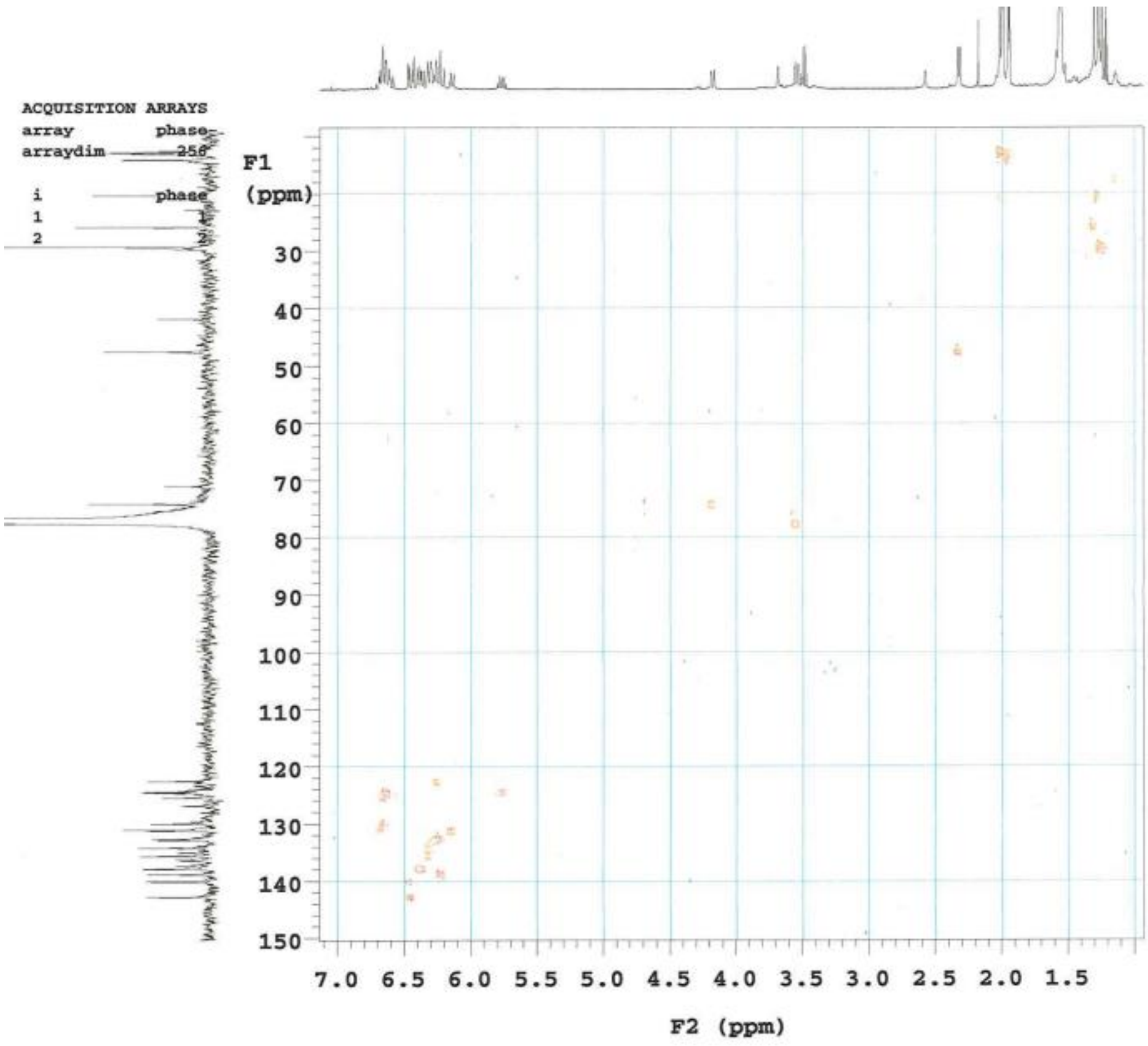

Fig. S12 HSQC spectrum of purified Peak III

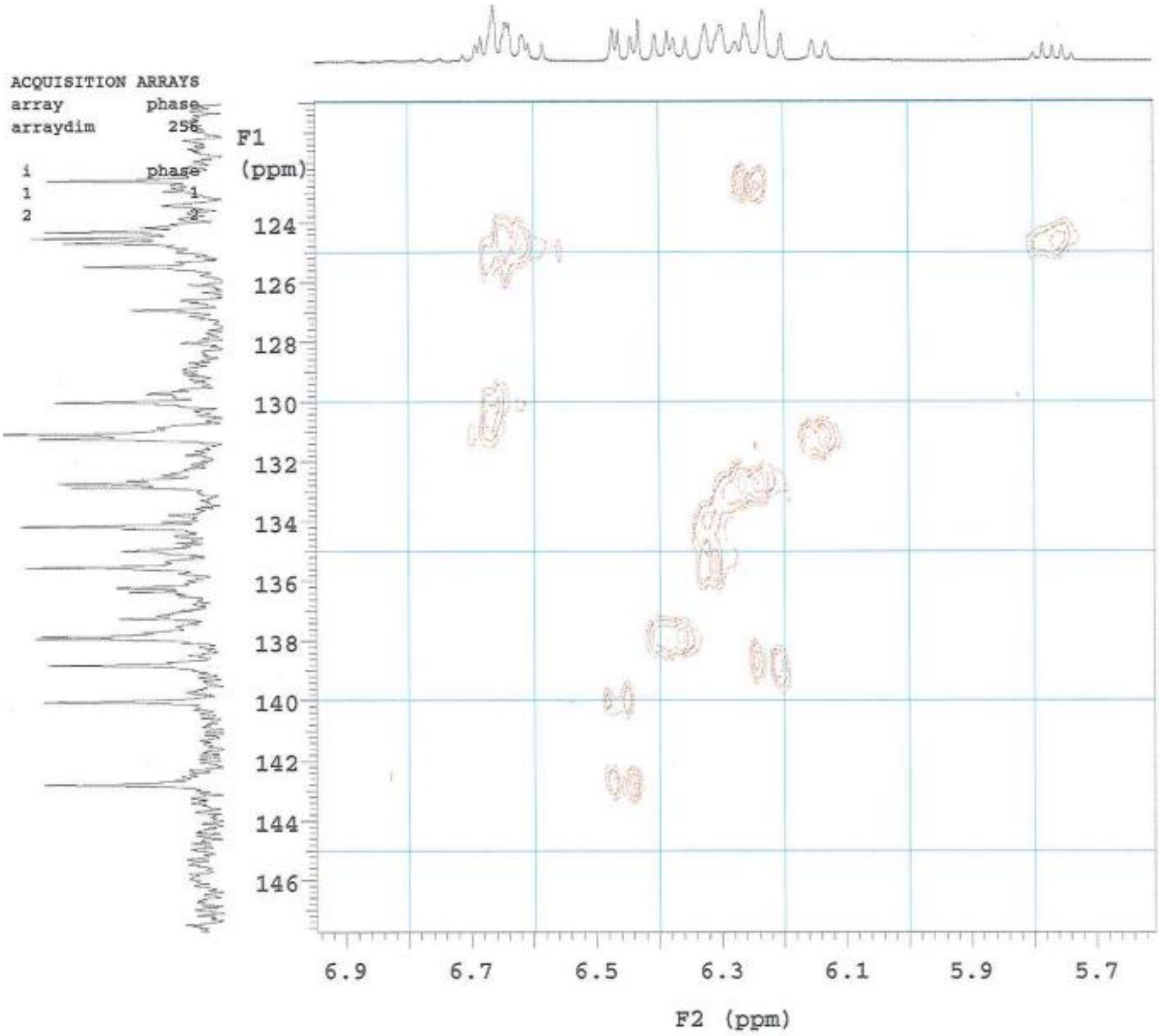

Fig. S13 Enlarged view of HSQC spectrum in Figure S12

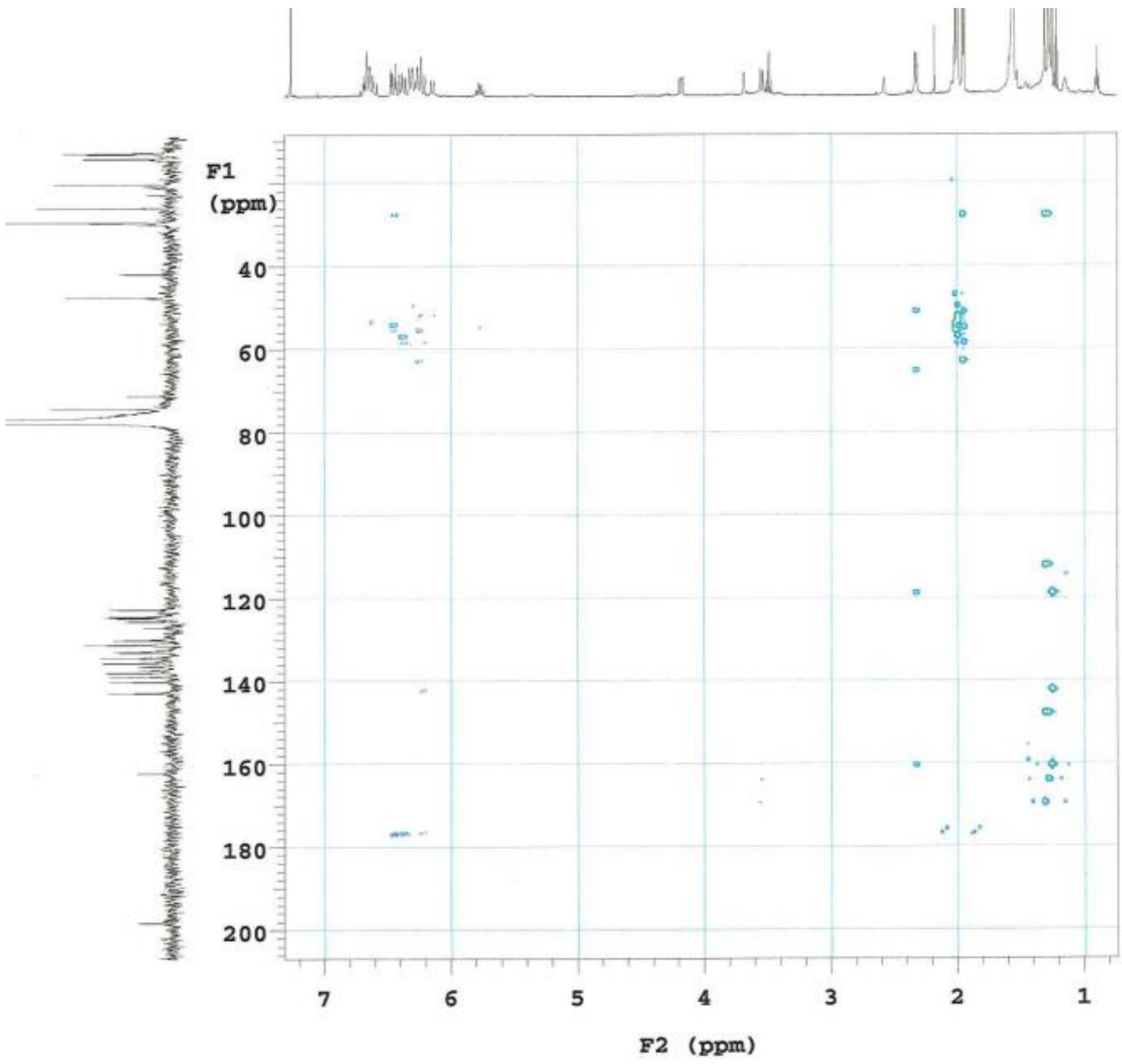

Fig. S14 HMBC spectrum of purified Peak III

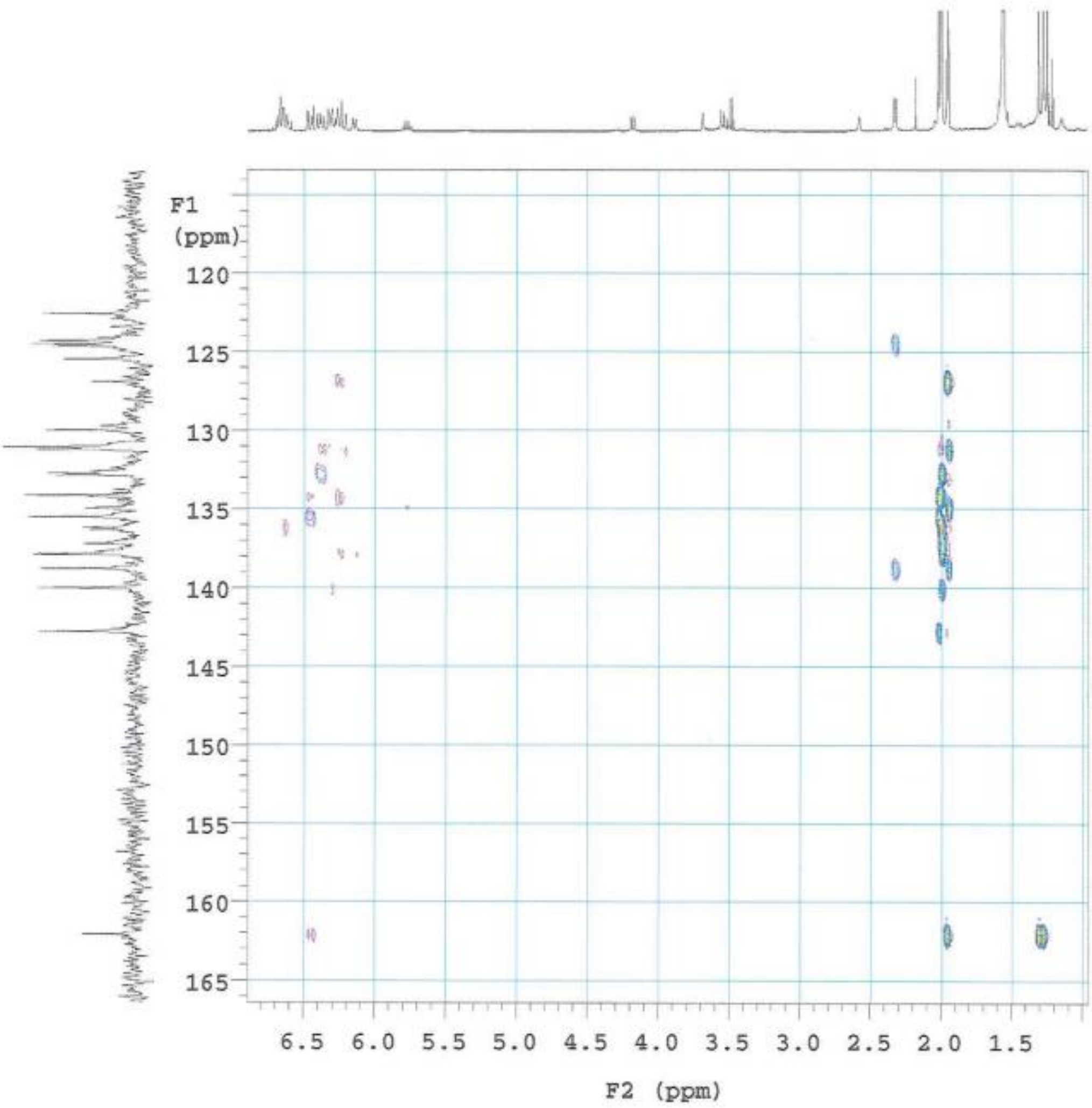

Fig. S15 Enlarged view 1 of HMBC spectrum in Figure S14

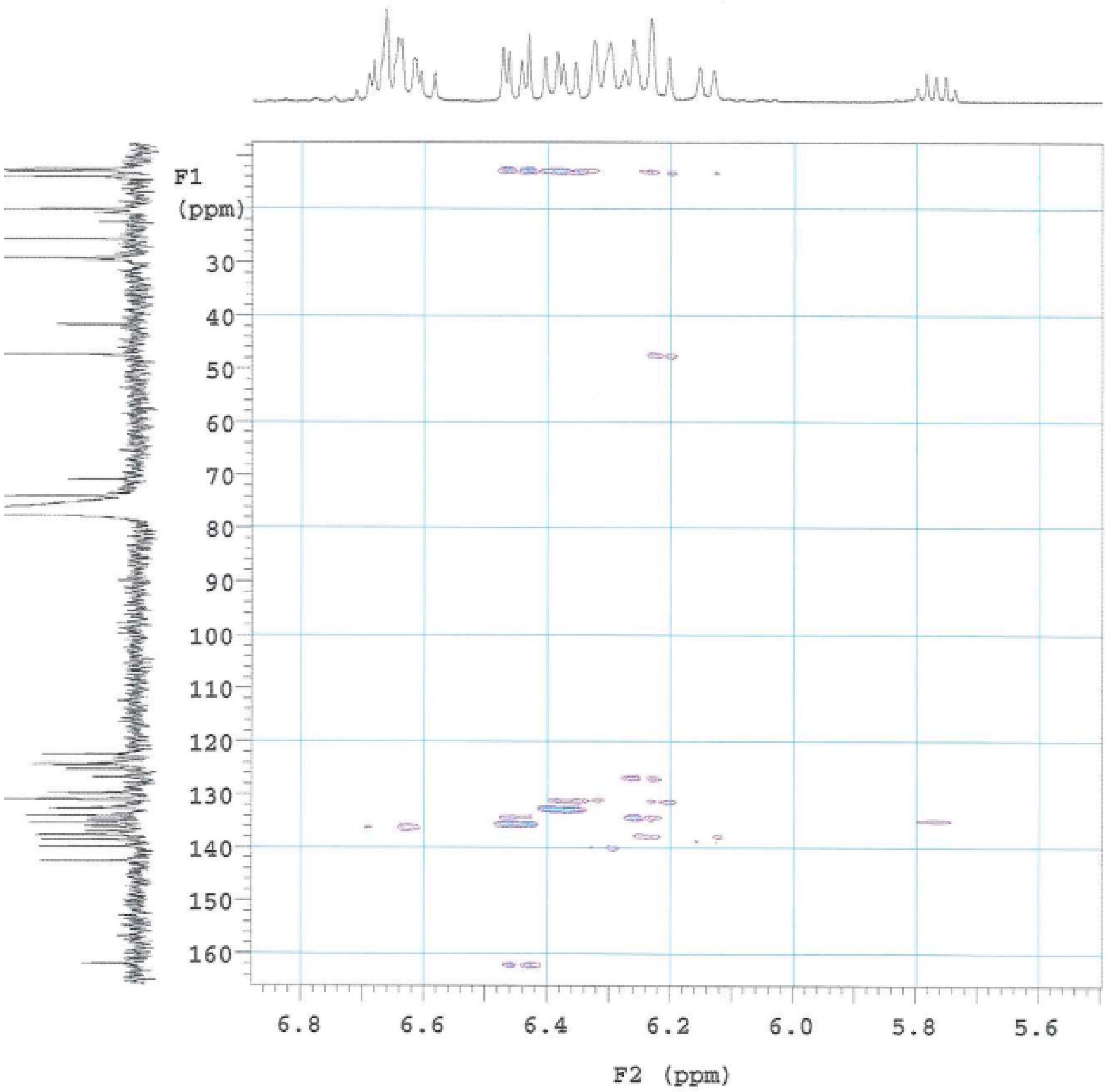

Fig. S16 Enlarged view 2 of HMBC spectrum in Figure S14

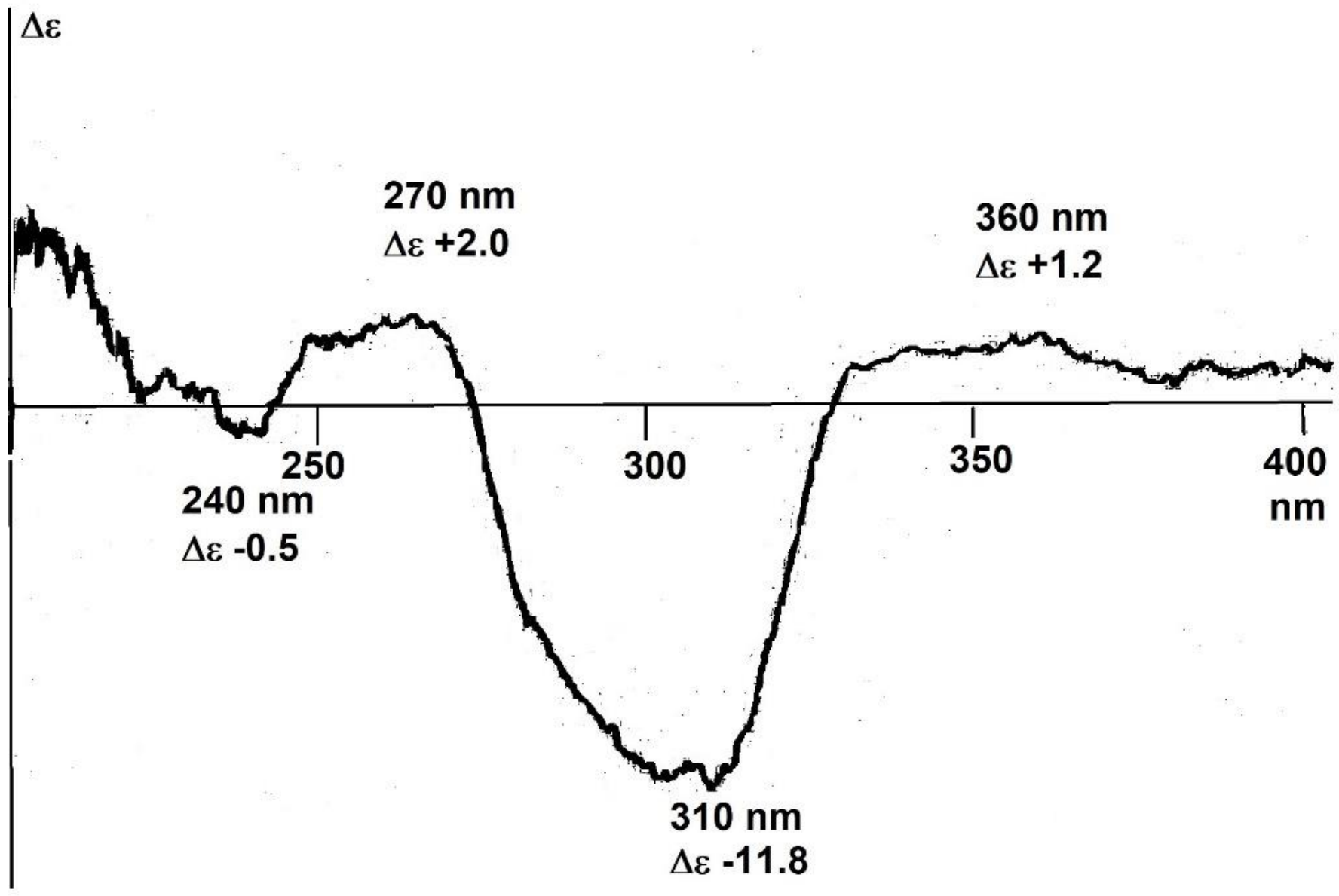

Fig. S17 CD spectrum of Purified peak III

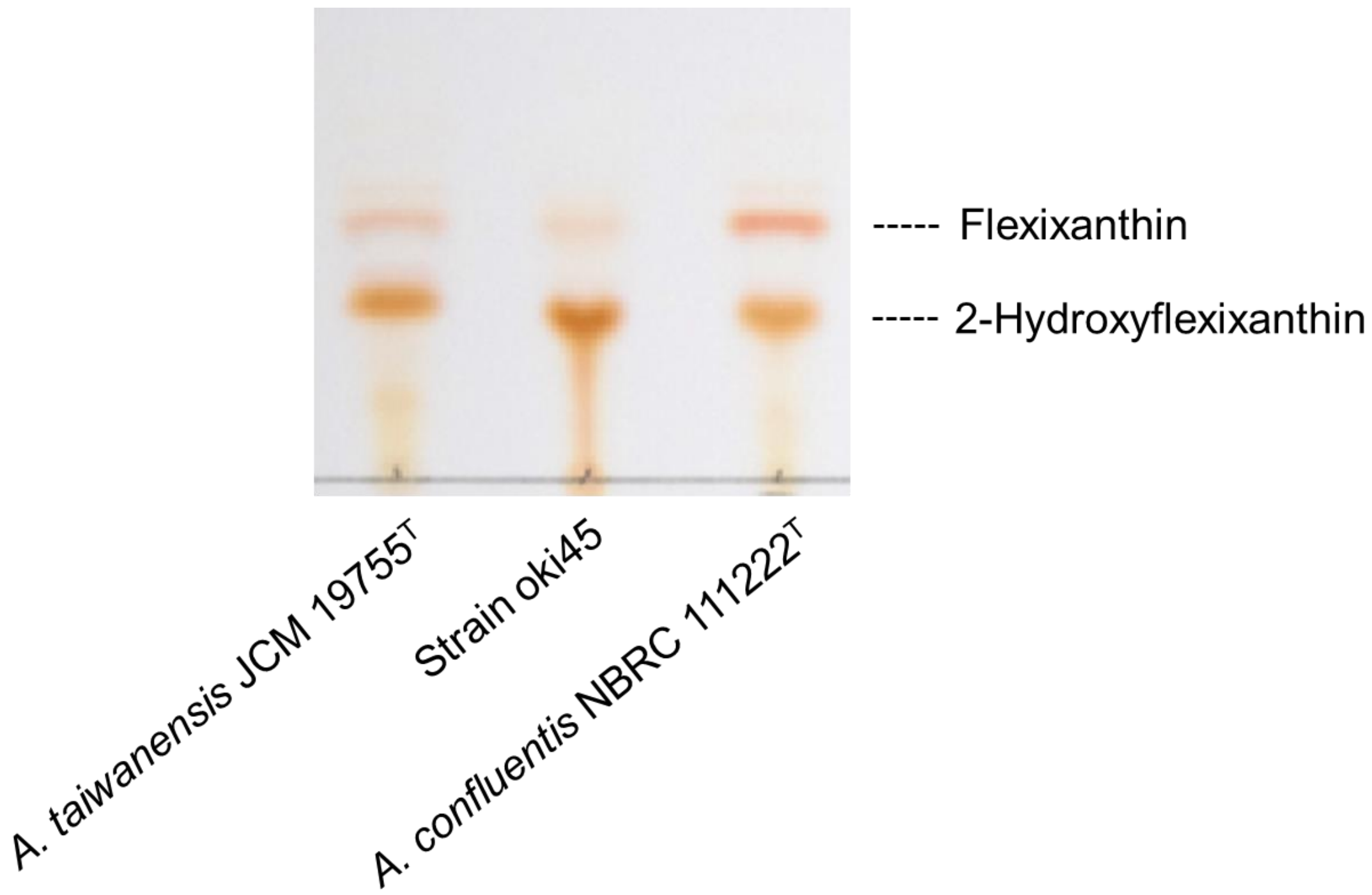

Fig. S18 TLC analysis of *Algoriphagus confluentis* NBRC 111222^T and *Algoriphagus taiwanensis* JCM 19755^T

Total lipids of three strains were extracted by Folch method and performed by n-hexane/acetone (60:40, v/v) on TLC plate with silica gel 60.

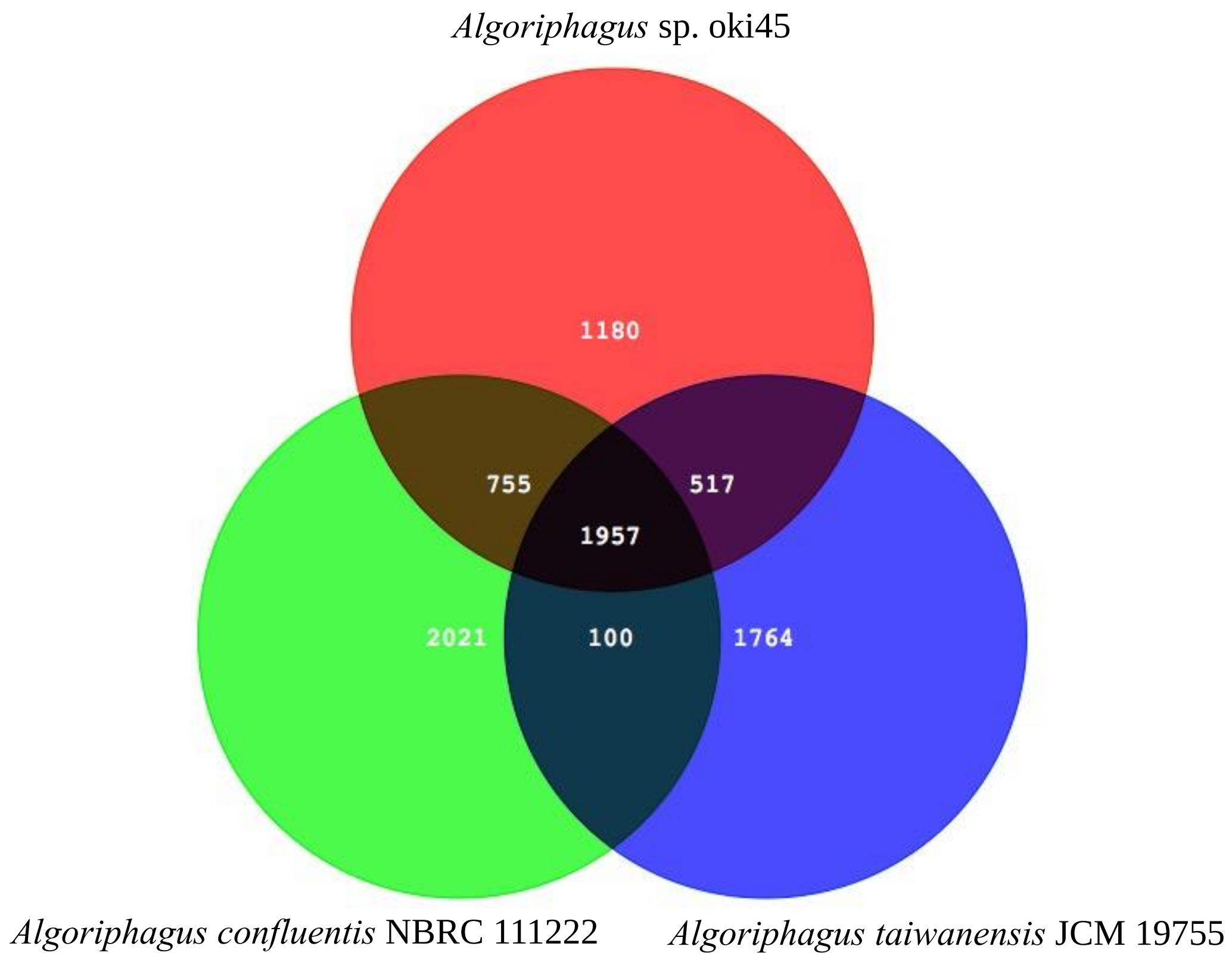

Fig. S19 Venn-Diagram comparing the genomic content of three *Algoriphagus*

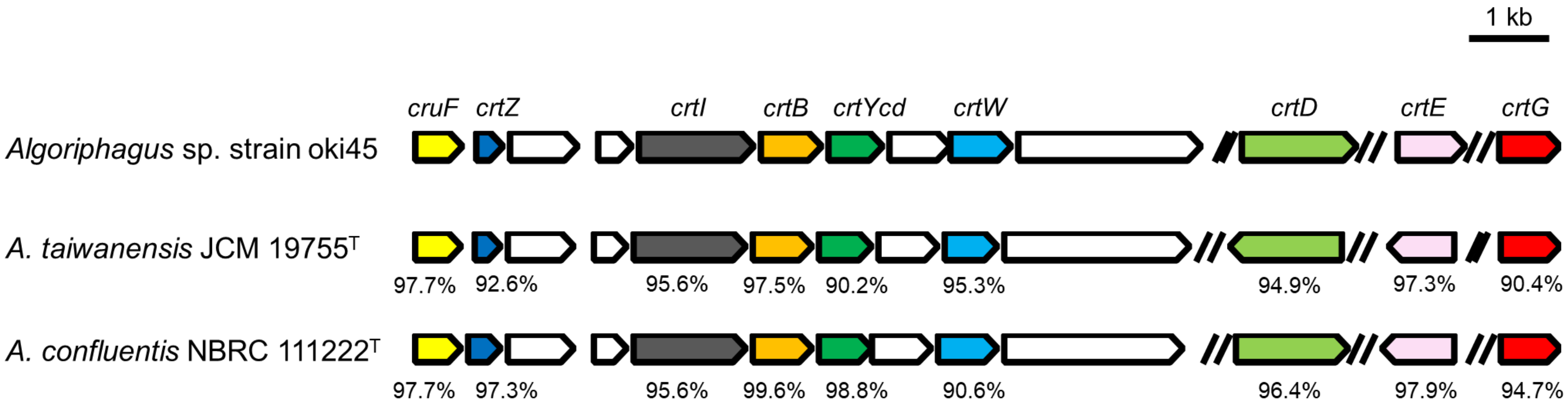

Fig. S20 Putative carotenoid biosynthetic genes in strain oki45, *A. confluentis* NBRC 111222^{T}, *A. taiwanensis* JCM 19755^{T}

Yellow, *cruF*; Blue, *crtZ*; Gray, *crtI*; Orange, *crtB*; Green, *crtYcd*; Light blue, *crtW*; Light green, *crtD*; Pink, *crtE*; Red, *crtG*.

Percentages in the figure indicate amino acid identity to that of the strain oki45.

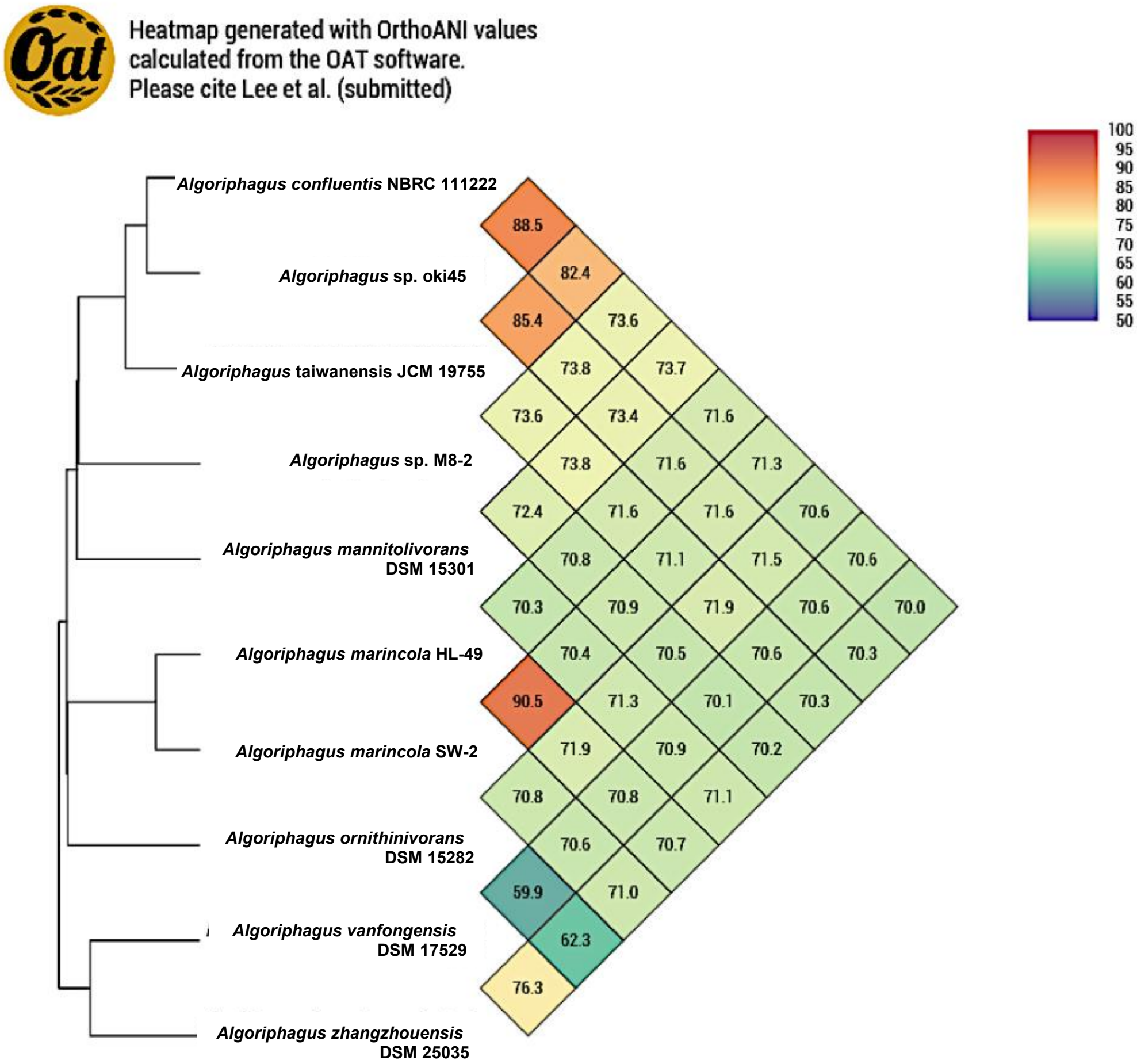

Fig. S21 Comparison of ANI between 10 *Algoriphagus*

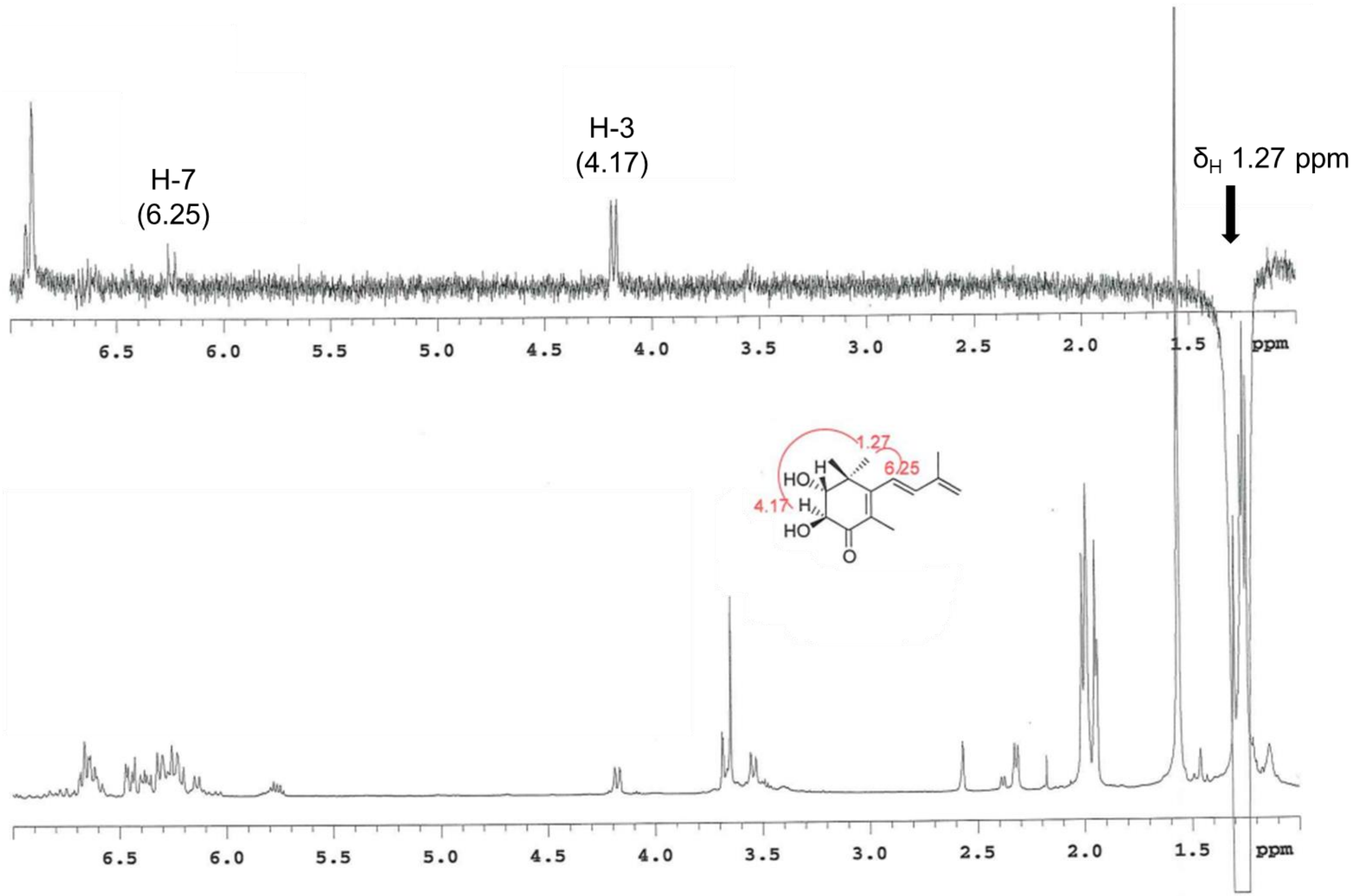

Fig. S22 1D NOE spectrum of purified Peak III (1)

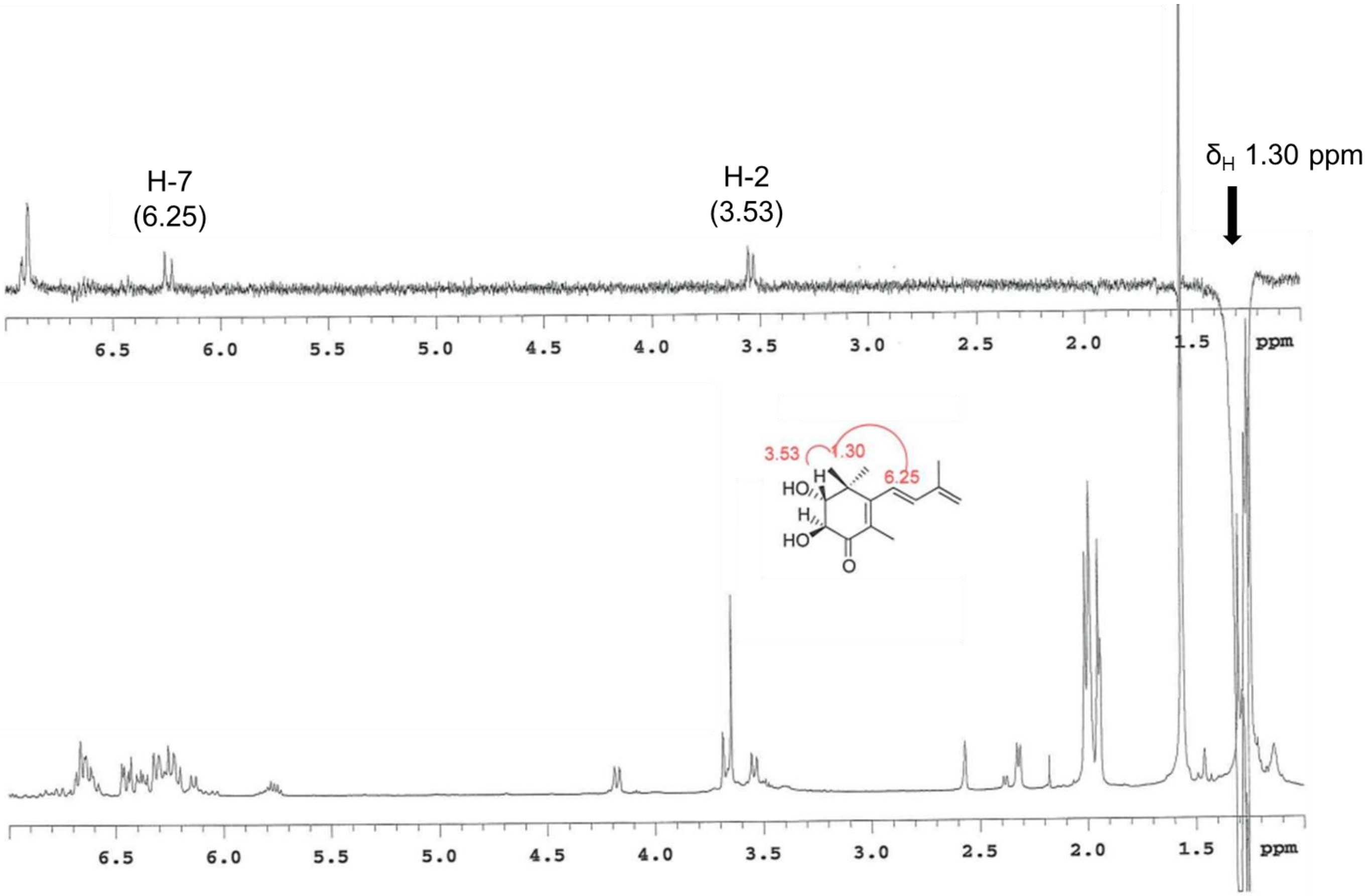

Fig. S23 1D NOE spectrum of purified Peak III (2)

Table S1 Calculation results using GGDC for three strains of oki45, *A. confluentis* NBRC 111222^T, *A. taiwanensis* JCM 19755^T

Query genome	Reference genome	DDH	Model confidence intervals	Distance	Probability that DDH >= 70%	G+C difference
Algoriphagus sp. oki45	*Algoriphagus confluentis*	35.7	[33.3 - 38.3%]	0.1151	0.83	0.24
Algoriphagus sp. oki45	*Algoriphagus taiwanensis*	29.5	[27.2 - 32%]	0.1447	0.09	0.1
Algoriphagus confluentis	*Algoriphagus taiwanensis*	25.2	[22.9 - 27.7%]	0.1725	0.01	0.14
